# Computational analysis of the solvation of coffee ingredients in aqueous ionic liquid mixtures†
†Electronic supplementary information (ESI) available. See DOI: 10.1039/c6ra24736a



**DOI:** 10.1039/c6ra24736a

**Published:** 2017-01-13

**Authors:** Veronika Zeindlhofer, Diana Khlan, Katharina Bica, Christian Schröder

**Affiliations:** a University of Vienna , Faculty of Chemistry , Department of Computational Biological Chemistry , Währingerstraße 19 , 1090 Vienna , Austria . Email: christian.schroeder@univie.ac.at ; Tel: +43 14277 52711; b Institute of Applied Synthetic Chemistry , Vienna University of Technology , Getreidemarkt 9/163 , 1060 Vienna , Austria

## Abstract

In this paper, we investigate the solvation of coffee ingredients including caffeine, gallic acid as representative for phenolic compounds and quercetin as representative for flavonoids in aqueous mixtures of the ionic liquid 1-ethyl-3-methylimidazolium acetate [C_2_mim][OAc] at various concentrations. Due to the anisotropy of the solutes we show that classical Kirkwood–Buff theory is not appropriate to study solvation effects with increasing ionic liquid content. However, excess coordination numbers as well as the mean residence time of solvent molecules at the surface of the solutes can be determined by Voronoi tessellation. Since the volume of the hydration shells is also available by this method, solvation free energies will be discussed as a function of the ionic liquid concentration to yield a physical meaningful picture of solvation for the anisotropic solutes. Hydrogen bonding capabilities of the solutes and their relevance for experimental extraction yields from spent coffee grounds are also discussed.

## Abstract 2

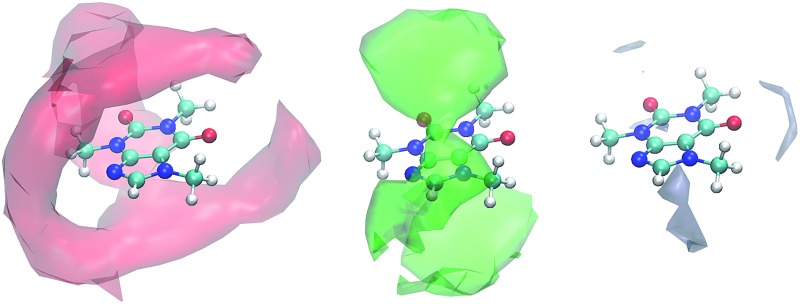
We investigate the solvation behavior of valuable coffee ingredients in aqueous mixtures of the ionic liquid 1-ethyl-3-methylimidazolium acetate with a particular emphasis on hydrotropic theory and Kirkwood–Buff analysis.

## 1 Introduction

**Fig. 1:**
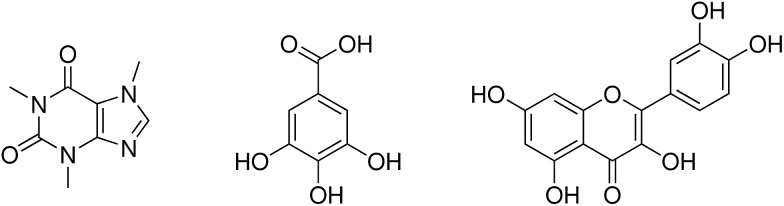
Caffeine (left), gallic acid and quercetin (right).

Coffee is a popular beverage brewed from roasted coffee beans. While coffee beans are among the leading export products from developing countries and production is still increasing,^1^ they do not only possess economic significance: physiological effects of several coffee ingredients have attracted considerable scientific interest.^2^ Among these, especially the main active component caffeine (1,3,7-trimethylxanthine, see Fig. 1) – which is also prominent in tea – is well-known for its beneficial and harmful impacts on the central nervous, muscular and cardiovascular system.^3–10^ In general, caffeine is a fairly hydrophobic molecule with limited solubility of 16 mg mL^–1^ in water. Only two carbonyl oxygens and the non-methylated nitrogen of the imidazole subunit (see Fig. 1) interact weakly with water, and hence caffeine tends to considerable self-association in aqueous medium.^11–15^

However, caffeine is not the sole active ingredient in coffee beans with physiological impact. Coffee phenolics gained interest based on their strong antioxidant activity and their metal-chelating properties. Moreover, many phenols in coffee have considerable biological activity against chronic diseases such as cataracts, as well as cancer and cardiovascular diseases.^16,17^ The total phenol content is often characterized in gallic acid equivalents,^16,18,19^ although, strictly speaking, gallic acid (3,5-dihydroxybenzoic acid, see Fig. 1) is not a natural coffee ingredient but can be found in various tea products.^20^ It has important properties in the health and nutrition fields because of its antibacterial, anti-fungal, anti-oxidative, phytotoxic and radical scavenging activities.^16,21,22^ However, gallic acid shows only low solubility of 12 mg mL^–1^ in water^21,23^ although all hydroxy groups are able to form hydrogen bonds to polar solvents.^24^

Quercetin (2-(3,4-dihydroxyphenyl)-3,5,7-trihydroxy-4*H*-1-benzopyran-4-on), another important phenolic compound found in coffee, is a naturally available flavonoid with an estimated human consumption of 1 g per day.^17^ Flavonoids exhibit anti-inflammatory and anti-allergic properties, anti-hepatotoxic, anti-fertility and anti-tumor activity,^17^ and recent studies showed that quercetin is the major neuroprotective component present in coffee.^25^ The total flavonoid content can be characterized in quercetin equivalents.^16^ Quercetin is almost insoluble in water^26,27^ with a solubility of less than 10 mg L^–1^ at 20 °C.^28^ Hydrogen bonding of the five hydroxy groups was only observed to lipid bilayers.^29^

These functional coffee ingredients are still present in spent coffee grounds after coffee brewery. Yet, spent coffee grounds are so far disposed as solid waste as they have no commercial value. Extraction of spent grounds would therefore provide an alternative source for a number of high-value health-related compounds while valorizing coffee production waste prior to its disposal.^16^ As a consequence, several recent studies address the extraction and quantification of caffeine, phenol compounds, flavonoids and other value-added ingredients from waste coffee using various solvents or solvent mixtures.^16,18,19,30^

The low solubility of valuable coffee ingredients in water is often compensated by the addition of alcohols. In 2011 Mussatto *et al.* investigated the extraction of active ingredients from spent coffee grounds with methanol as a solvent.^16^ The maximum value of phenols was extracted from spent coffee grounds with water/methanol solutions at 50% v/v after 90 min extraction time at 60 °C to 65 °C. Similarly, the flavonoid content could be almost tripled when water/methanol solutions where used instead of pure water.^16^ Also several experimental studies concern the extraction of caffeine^30–33^ and gallic acid^21,23,30,34,35^ with ionic liquid/water mixtures exploiting the amphiphilic character of various ionic liquids.^36,37^ These aqueous mixtures are also appealing since they reduce the prize issue of ionic liquids.^30,37^ In 2013, Cláudio *et al.* investigated the extraction of caffeine from guarana seeds^32^ using ionic liquid/H_2_O. The caffeine extraction yield of 3.86 wt% in pure water was gradually increased to 8.18 wt% by adding 1-butyl-3-methylimidazolium chloride from 0.5 M to 3 M. In particular, the imidazolium cation seems to play an important role since the addition of 1 M NaCl to the aqueous mixture decreases the extraction yield of caffeine. However, at 1.5 M the original extraction yield of caffeine in water is almost regained.

In this study, we will analyze the solvation behavior of caffeine, gallic acid (representing coffee phenolics) and quercetin (representing flavonoids) in aqueous mixtures of 1-ethyl-3-methyl-imidazolium acetate [C_2_mim]OAc at ionic liquid concentrations of 0 M to 6.5 M.

## 2 Theory

### 2.1 Hydrotropic effect

The benefit of a co-solvent for the solubility of hydrophobic solute in aqueous solution is also discussed theoretically in terms of the hydrotropic effect: the hydrophobic solvation is enhanced by hydrotrope-induced water activity depression^38^ and/or accumulation of the hydrotrope around the solute promoting the solubilization by several orders of magnitude.^38,39^ This phenomenon is similar to the “salting-in” effect observed for the solvation of proteins.

Hydrotropes are usually highly soluble in water and they possess an amphiphilic structure and a significant surface activity to bind to a hydrophobic solute.^40,41^ In principle, three possible mechanisms are suggested, which are not mutually exclusive:^23,38,42–44^

(1) Self-aggregation of the co-solvent around the solute at typical concentrations of 1–10 mM which solubilizes the solute. Although the concentration behavior is similar to micelles consisting of surfactants,^38,40^ hydrotrope-solute aggregates does not necessarily involve a complete covering of the solute. Furthermore, hydrotropes differ from surfactants by a much higher hydrophile/lipophile balance.^23,42^ However, this mechanism is not very likely for amphiphilic molecules with short alkyl chains which need much higher concentrations to form micelle-like structures.^45^

(2) The formation of persistent solute–hydrotrope complexes with low stoichiometry or co-aggregation necessitates significant hydrophobic interactions between the partners, *e.g.* π–π stacking of planar aromatic rings or strong van-der-Waals interactions.^23,38,44,46^ In addition, the hydrotrope should also consist of several polar moieties to interact with water,^40^ for example as hydrogen bond donor or acceptor.^46^ As a result, the hydrotrope acts as mediator between the hydrophobic solute and water.^38,47^ However, there also exist examples of non-stoichiometric aggregates.^48^

(3) The hydrotrope does not bind directly to the solute but acts as structure-breaker for water. It counteracts the freezing water structure around the hydrophobic solute and prevents the entropy decrease.^38,49–51^ It also may alter the probability of water hydrogen bonding.^48,52^ Molecular hydrotropes using the last two mechanisms usually operate at concentrations of 1–3 M.

Imidazolium based ionic liquids would in principle fulfill the hydrotrope criteria since the cations possess polar domains like their charged ring as well as apolar regions like the alkyl side chains. By proper choice of the anion, the water solubility can be tuned. Furthermore, imidazoliums with long alkyl chains are known to form micelles in water,^53^ which can accommodate hydrophobic solutes. However, many short chain imidazolium based ionic liquids have been investigated for their hydrotropic potential by Coutinho and co-workers.^23^ They found that the π–π stacking cannot not be the sole source for hydrophobic interactions since some of the non-aromatic ionic liquids significantly increased the solubility of gallic acid and vanilin. Furthermore, the hydrotropic effect seems to be solute specific as different trends for the two above mentioned solutes are found. Although the hydrotropic behavior of many cation/anion combinations were studied in ref. 23, [C_2_mim]OAc was not investigated despite its potential for the extraction of valuable ingredients from biomass.^54^ Consequently, we would like to study the hydrotropic potential of [C_2_mim]OAc for caffeine, gallic acid and quercetin.

### 2.2 Kirkwood Buff theory

The three proposed mechanisms of hydrotropy can be analyzed in terms of the classical Kirkwood–Buff theory.^38,43,55–59^ The self-aggregation of the co-solvent at the surface of the solute leads to positive Kirkwood–Buff integrals1
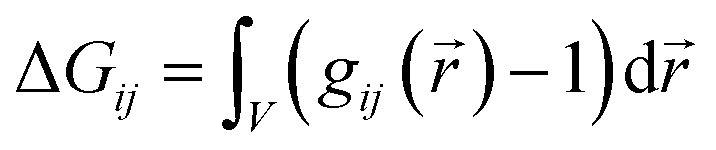
of species *j* around *i*. Negative values of Δ*G*_*ij*_ indicate less favorable binding of the solvent species. Computational studies on hydrotropic effects using Kirkwood–Buff are already reported.^38,39,44^ These data can be compared to corresponding experimental data.^55,58^

However, in literature^14,44,55–57,59–62^ the volume integration in eqn (1) is usually performed *via* spherical shells2
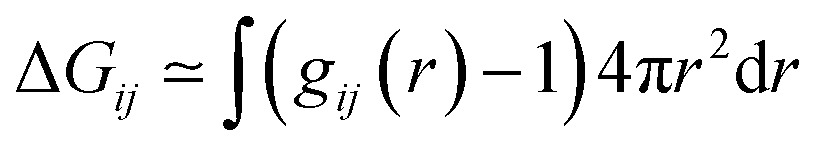
for the sake of simplicity. The integration starts at the surface of the solute and ends a value where Δ*G*_*ij*_ has converged. However, the convergence behavior is usually poor.^63^

**Fig. 2:**
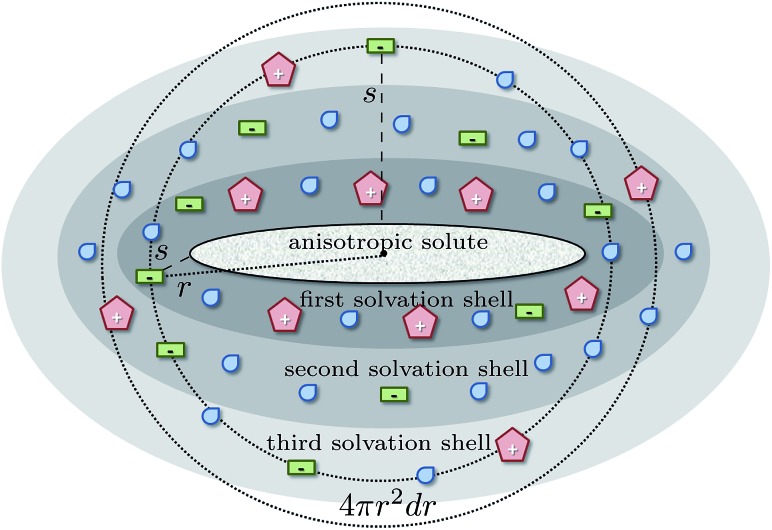
Schematic view on the solvation of an anisotropic solute and arising issues of a spherical analysis in terms of radial distribution functions *g*_*ij*_(*r*).

The spherical integration assumes an isotropic solute and may lead to dubious results in case of anisotropic solutes as sketched in Fig. 2. At a certain distance *r* from the center of mass of the anisotropic solute molecule the integration of the radial distribution function according to eqn (2) includes solvent molecules in the first solvation shell (dark gray), second solvation shell (gray) and even third solvation shell (light gray). Consequently, the solvent structure of these molecules is averaged and does not reflect the solvation at the surface of the solute. In principle, Song *et al.* ^64^ circumvented this problem by calculating effective local densities as a function of the distance *s* of a solvent molecule to the nearest solute atom instead of the distance *r* between the respective center of masses. However, although this distance reflects the solvation shells in Fig. 2 better, the respective radial distribution function *g*_*ij*_(*s*) still has to be normalized for the volume of the corresponding spherical shell *V* = 4π*r* ^2^d*r* which may lead to some problems, *e.g.* if parts of this volume are excluded by the solute. Nevertheless, *g*_*ij*_(*s*) should be superior compared to *g*_*ij*_(*r*) for the analysis of structural changes of the solvent water around the solute when increasing the concentration of the co-solvent as proposed by the third mechanism of hydrotropy.

Restricting the Kirkwood Buff integral to the first solvation shell and multiplying the corresponding Δ*G*_*ij*_ by the respective solvent density *ρ*_*j*_ yields excess coordination numbers,^15,39,65,66^ ΔCN_*j*_ = CN_*j*_ – (4π/3)*R* ^3^ *ρ*_*j*_, which are also used to characterize solvation properties, like dewetting.^39,43^ Here, we have used the cumulative coordination numbers CN_*j*_ of solvent species *j*
3
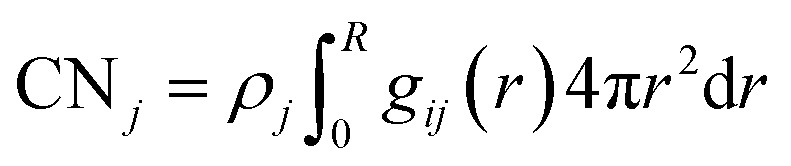
gained by spherical integration up to the shell thickness *R* (usually approximated by the first minimum of *g*_*ij*_(*r*)). However, in case of anisotropic solute molecules, solvent molecules of the second and third solvation shell contribute to CN_*j*_. In particular, for almost planar solutes like our coffee ingredients in Fig. 1 solvent molecules above and below the aromatic ring systems may count to CN_*j*_ at a certain distance *R* although not belonging to the first solvation shell, whereas solvent molecules with a hydrogen bond to the solute hydroxy groups are far away from the solute center of mass and may be not within the distance *R*. Furthermore, it is not so easy to determine a particular value for the upper integration limit *R* in case of flat *g*_*ij*_(*r*).

Consequently, we applied a different approach, the so-called Voronoi tessellation,^67,68^ which is parameter free. Here, the complete space is decomposed into irregular polyhedra. Each of these polyhedra contains all points in space closer to the reference molecule than to any other molecule. Direct neighbors can be easily detected since their polyhedra share a face. Molecules in the second solvation shell are neighbors of the first neighbors and so on.

The formation of solute/co-solvent complexes, as inferred by the second hydrotropy mechanism, can be studied by the mean residence time of molecules in the first solvation shell. Based on the Voronoi tessellation, the residence function *n*_*j*_(*t*)4
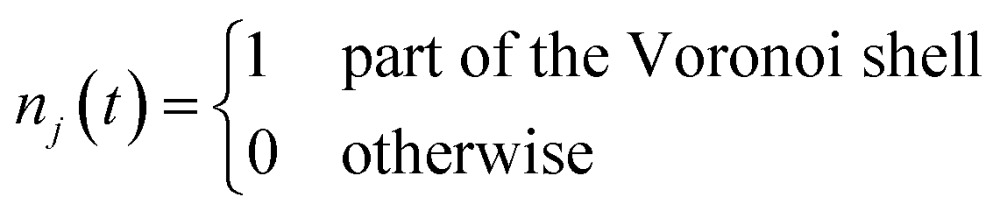
determines the sojourn of a particular molecule of solvent species *j* at the solute surface. In order to avoid problems with periodic boundary conditions, only members of the first Voronoi shell are taken into account which have not left the primary simulation box after re-centering around the respective solute at each time step. The first value of the auto-correlation function *n*_*j*_(0)·*n*_*j*_(*t*) yields the coordination number CN_*j*_ = *n*_*j*_(0)^2^ and the relaxation time equals the mean residence time of that solvent species which characterizes the life time of solute/co-solvent complexes.

## 3 Methods

### 3.1 Molecular dynamics simulations

The force field of caffeine, gallic acid and quercetin was obtained by Swissparam ^69^ and used without any further modifications. The force field parameters are listed in the ESI.† Since we are interested in aqueous mixtures of C_2_mim acetate, the force field of this ionic liquid was generated by Gaamp ^70^ which explicitly uses the interaction with water to develop the force field parameter which can also be found in the ESI.† In contrast to the well-known parametrization of Canongia Lopes and Pádua^71^ the absolute charges of the carbons and hydrogens of the aliphatic chain are a little bit smaller and the net charge of the imidazolium ring is about 10% less. The Lennard-Jones parameter of the carbons have higher *ε* but lower *σ* values, the trend for the hydrogens is opposite. The dihedral rotation of the ethyl chain was re-parametrized. This changes seem necessary since the parametrization of imidazolium in ref. 71 only allows for very weak hydrogen bonding^72^ which may be important for aqueous mixtures investigated here and plays a significant role for the extraction of biomolecules.^54^ Our parametrization of acetate is similar to OPLS-AA^73^ which is also used by Canongia Lopes and Pádua. Water was modeled by the SPC/E model of Berendsen *et al.* ^74^

An overview on the manifold of independent molecular dynamics simulations is given in Table 1. Each simulation contains only one solute which corresponds to a concentration of roughly 8–12 mM. The box size *l* in Table 1 was obtained by an npT simulation of 4 ns at *T* = 300 K and atmospheric pressure performed with CHARMM.^75^ The cut-off radius for the long-range interactions was set to 14 Å and an Ewald parameter of *κ* = 0.41 Å^–1^ was used. After this equilibration, the box length was fixed and a nVT simulation of each system with a time step of Δ*t* = 2 fs was performed at *T* = 300 K for 20 ns. Since the water diffusion coefficients, *D*_H_2_O_, in Table 1 depend only on the concentration of the ionic liquid but not on the nature of the solute, the simulation boxes are large enough so that the diffusion coefficients are governed by bulk water molecules which outnumber water molecules at the surface of the respective coffee ingredient.

**Table 1:** Composition of the simulation boxes containing one solute and the corresponding numbers of [C_2_mim][OAc]/water. The corresponding ionic liquid concentration *c*_IL_ is given in mole per liter (M) and weight percent (wt%). *D*_H_2_O_ is the water diffusion coefficient

Solute	Ion pairs	Water	*l* [Å]	*c*_IL_		*D*_H_2_O_ [Å^2^ ps^–1^]
				[M]	[wt%]	
Caffeine	—	7188	60.015	—	—	0.27
	100	4160	52.940	1.1	18	0.16
	200	3320	52.740	2.3	36	0.079
	400	1600	52.550	4.6	70	0.004
	842	—	60.124	6.5	100	—
Gallic acid	—	7188	60.007	—	—	0.27
	100	4160	52.930	1.1	18	0.17
	200	3320	52.720	2.3	36	0.079
	400	1600	52.520	4.6	70	0.004
	842	—	60.075	6.5	100	—
Quercetin	—	7188	60.021	—	—	0.27
	100	4160	52.950	1.1	18	0.17
	200	3320	52.750	2.3	36	0.080
	400	1600	52.570	4.6	70	0.004
	842	—	60.021	6.5	100	—

### 3.2 Experimental extraction

#### 3.2.1 General

Commercially available reagents and solvents were purchased from Sigma Aldrich unless otherwise specified. [C_2_mim]OAc was obtained from BASF (Germany). For biomass preparation 1 kg of spent coffee grounds was collected from 100% Arabica (Illy espresso) and dried in a vacuum drying oven at 60 °C/20 mbar for two weeks, until there was no change of mass.

High performance liquid chromatography (HPLC) analysis was performed on Jasco HPLC unit equipped with a PDA detector. Ultraviolet-visible light spectroscopy (UV-VIS) was performed on a Shimadzu UV/VIS 1800, with wavelength range of 190 to 1100 nm, and spectral bandwidth of 1 nm. For the determination of Caffeine a Maisch Reprosil 5 μm C18 column (250 × 4.60 mm) was used with Methanol/H_2_O/5% trifluoroacetic acid 25/75 as solvent and a flow of 1 mL min^–1^; detection was done at 210 nm. Calibration curves were in the range of 2.0–0.005 mg mL^–1^ using phenol as internal standard (50 mg/100 mL MeOH). Retention times were 16 min for caffeine and 20 min for the internal standard phenol.

Total phenol content of extraction samples was analyzed by means of a colorimetric method using the Folin–Ciocalteu reagent. Stock solutions of gallic acid for the calibration of the total phenol content were prepared in range of 3–0.2 mg mL^–1^ in water or in water : [C_2_mim]OAc (10 : 90) mixtures.

Flavonoid content of extraction samples was determined by UV-Vis spectroscopy using quercetin as standard. Stock solutions of quercetin for the calibration of the total flavonoid content were prepared in range of 0.2–0.025 mg mL^–1^ in water or in water:[C_2_mim]OAc (10 : 90) mixtures.

#### 3.2.2 Extraction of spent coffee grounds

A 5 mL screw-cap vial was charged with a 10 wt% of spent coffee grounds (0.100 ± 0.010 g) in the respective solvent (0.900 ± 0.150 g) and stirred at room temperature for 24 h. The suspensions were diluted to 5 mL using ethanol. For the analysis of caffeine content, a sample of 1 mL was immediately taken from the solution and 0.2 mL of internal standard solution (phenol) were added. The samples were centrifuged for 5 min at 13 000 min^–1^ and the supernatant solution was directly analyzed *via* HPLC. For the total phenol content analysis, a sample of 50 μL was from the solution of spent coffee grounds in ethanol, 600 μL of sodium carbonate solution (7.5% w/v), 150 μL of Folin–Ciocalteu reagent and 2000 μL of water were mixed and heated to 60 °C for 5 min. The samples were analyzed for determination of total phenol content by UV-VIS spectroscopy at a wavelength of 700 nm. For the total flavonoid analysis, a sample of 300 μL was taken from the solution of spent coffee grounds in ethanol, 900 μL of methanol, 60 μL of aluminum chloride (10% w/v), 60 μL of potassium acetate (1 mol L^–1^) and 1700 μL of water were added, mixed and stored for 30 min at room temperature in a dark place. The samples were analyzed for their content of flavonoids by UV-VIS spectroscopy at a wavelength of 415 nm. All experiments were repeated five times and the corresponding extraction yields are reported as mean value.

## 4 Results and discussion

### 4.1 Anisotropy issues

**Fig. 3:**
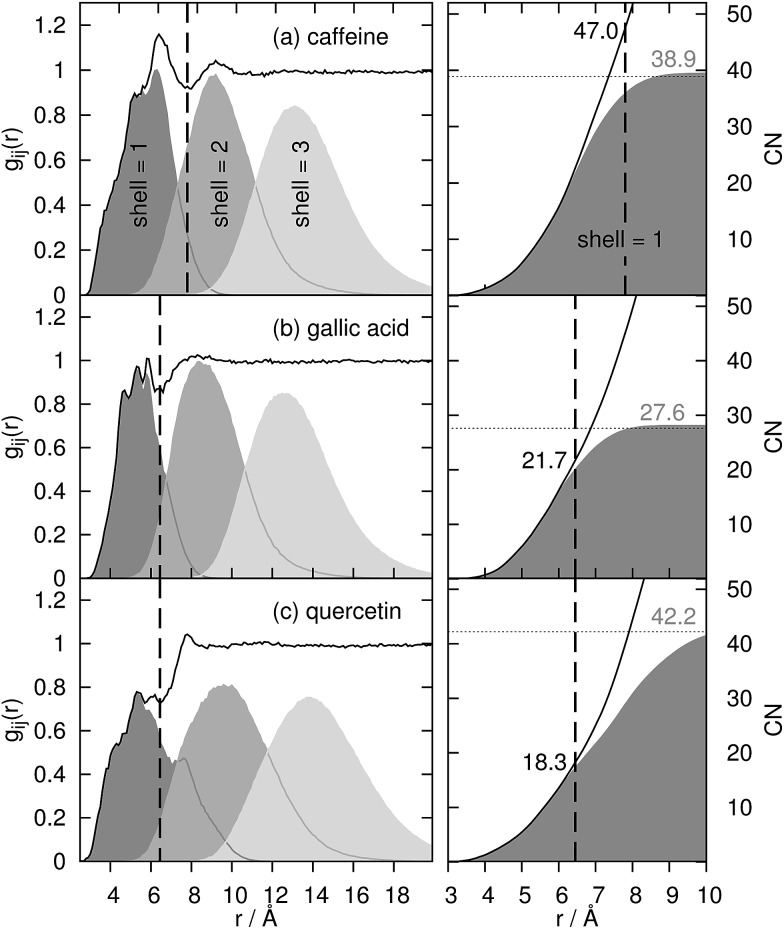
Radial distribution function *g*_*ij*_(*r*) of water around the solutes and its decomposition into first, second and third solvation shell (shaded areas). The black dashed line represents the distance of the “first minimum” of *g*_*ij*_(*r*) to determine the coordination number (black numbers at right column) *via* spherical integration.

For the coffee ingredients caffeine, gallic acid and quercetin, the radial distribution function *g*_*ij*_(*r*) (left graphs) is displayed in Fig. 3. The vertical, dashed line represents a tentative limit of the first solvation shell at a distance of *R* obtained by the common procedure from the first minimum of *g*_*ij*_(*r*) and result in *R* = 7.8 Å for caffeine and 6.45 Å for gallic acid and quercetin, respectively. This procedure is not without ambiguities as demonstrated by the determination of the “correct” shell thickness *R* in case of gallic acid and quercetin where several minima exist. In contrast, the shell assignment by means of the Voronoi residence function (*cf.*eqn (4)) is straightforward. For the dark gray shaded areas only those contributions to *g*_*ij*_(*r*) are counted which also possess *n*(shell = 1, *t*) = 1, *i.e.* the solvent molecule is in the first shell of the coffee ingredient. The gray and light gray shaded areas are made up by molecules which are in the second (*n*(shell = 2, *t*) = 1) and third (*n*(shell = 3, *t*) = 1) solvation shell. Of course, the original *g*_*ij*_(*r*) is regained as the sum of all solvation shell contributions since all molecules are assigned to a particular shell at time *t*.

Comparing the Voronoi decomposition of *g*_*ij*_(*r*) with the former method of the first local minimum, one immediately notices the overlapping of the solvation shells in Fig. 3. Between 8 and 10 Å the first and third shell overlap for all three solutes investigated here which corresponds to the situation sketched in Fig. 2. Consequently, using a single threshold *R* for the first solvation shell will result in disregarding some first solvation shell members (in particular near the hydroxyl groups far away from the center of mass of the solute) and taking into account some molecules from the second solvation shell below or above the aromatic ring systems.

This has significant consequences for the coordination number CN_*j*_ and its interpretation in Kirkwood Buff terms: using eqn (3) the cumulative integrand is depicted as solid black lines in the right graphs of Fig. 3. At the threshold *R* (vertical dashed line) coordination numbers CN_H_2_O_ of 47.0, 21.7 and 18.3 for caffeine, gallic acid and quercetin are gained. Using Voronoi tessellation CN_H_2_O_ = *n*_*j*_^2^ numbers of 38.9, 27.6 and 42.2 for caffeine, gallic acid and quercetin are obtained (as visible by the gray numbers in Fig. 3). Comparing the Voronoi coordination numbers with those estimated from eqn (3) reveals that the water coordination around caffeine is slightly overestimated by eqn (3) and slightly underestimated in case of gallic acid. The largest discrepancy occurs for quercetin which is also the most anisotropic molecule (see Fig. 1) and stems from disregarding the long tail of the dark shaded area in the left graph of Fig. 3c.

However, if one only uses the Voronoi contribution to *g*_*ij*_(*r*) of the first shell and performs the spherical integration in eqn (3) (dark gray areas at the right graphs) the Voronoi coordination numbers are regained as visible by the horizontal dashed lines. The distance where the spherical integration meets this number depends on the maximum extension of the first solvation shell. Since quercetin is the most anisotropic molecule, this distance is larger (≃10.5 Å) compared to 8 and 9 Å for gallic acid and caffeine.

### 4.2 Solvation free energy

**Fig. 4:**
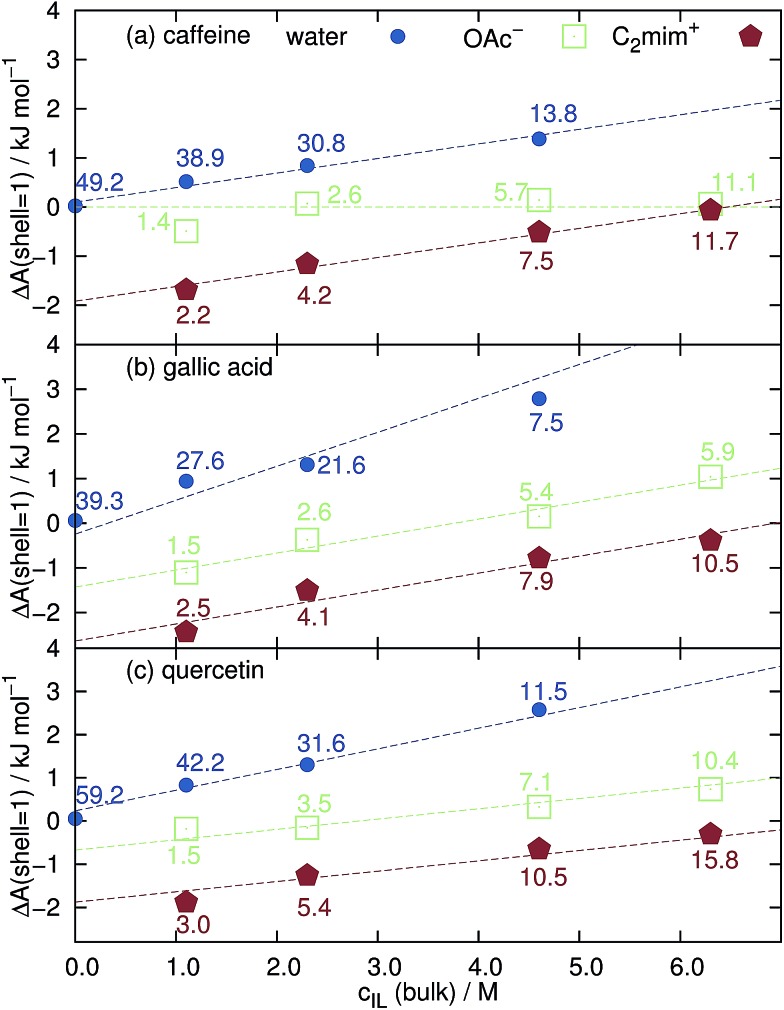
Free solvation energy Δ*A* of the first solvation shell around (a) caffeine, (b) gallic acid and (c) quercetin as a function of the bulk concentration of the ionic liquid. The numbers denote CN_*j*_ of the respective solvent species *j* in the first solvation shell.

Since the total volume of all Voronoi polyhedra in the each shell can be determined, it reflects the original idea of Kirkwood–Buff (see eqn (1)) and can also be used to compute concentrations *c*_*j*_(shell) = CN_*j*_(shell)/*V*(shell) of the solvent molecule *j* at the respective solvation layer. The solvation free energy Δ*A* is then5
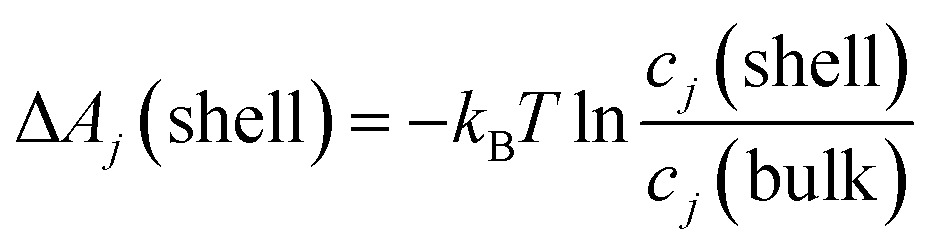
at the temperature *T* and *c*_*j*_(bulk) = *ρ*_*j*_/*N*_A_. Since the influence of the solute on the concentration of the solvent species *j* decreases with increasing distance, Δ*A*_*j*_(shell) approaches zero for the most distant solvation shells. Negative values at the first and second solvation shell indicate a preferred solvation. The expelling of a solvent species *j* at the solute surface, for example in case of dewetting, is reflected by positive Δ*A*_*j*_(shell) values. In pure water, first solvation shell Δ*A*(shell = 1)-values of hydrophobic caffeine, gallic acid and quercetin are very close to zero in Fig. 4 which means that the concentration of water at the surface of the solute is the same as in bulk. This is expected since water cannot be replaced by ionic liquid molecules at *c*_IL_ = 0 M and void volume is not possible within the Voronoi tessellation since it is automatically assigned to the nearest molecule.

However, increasing the concentration of [C_2_mim]OAc leads to a dewetting of the coffee ingredient as shown by the increasing positive values of Δ*A*(shell = 1) in Fig. 4. Interestingly, the solvation free energy of acetate around caffeine is almost independent of *c*_IL_ and close to zero. This may be due to the fact that caffeine possesses no hydroxyl groups which are favorable binding sites for the anions.^54^ The slopes (dashed lines in Fig. 4) of the cations and water in Fig. 4a look alike showing that the water molecules are expelled by the cations from the caffeine surface. In case of gallic acid and quercetin in Fig. 4b and c the slopes of cation and anion are equal and their sum corresponds to the slope of water. Here, both cations and anions remove water from the surface. Above *c*_IL_ = 4 M, the solvation free energy of the anions becomes positive indicating that the cations start to expel anions from the surface.

**Fig. 5:**
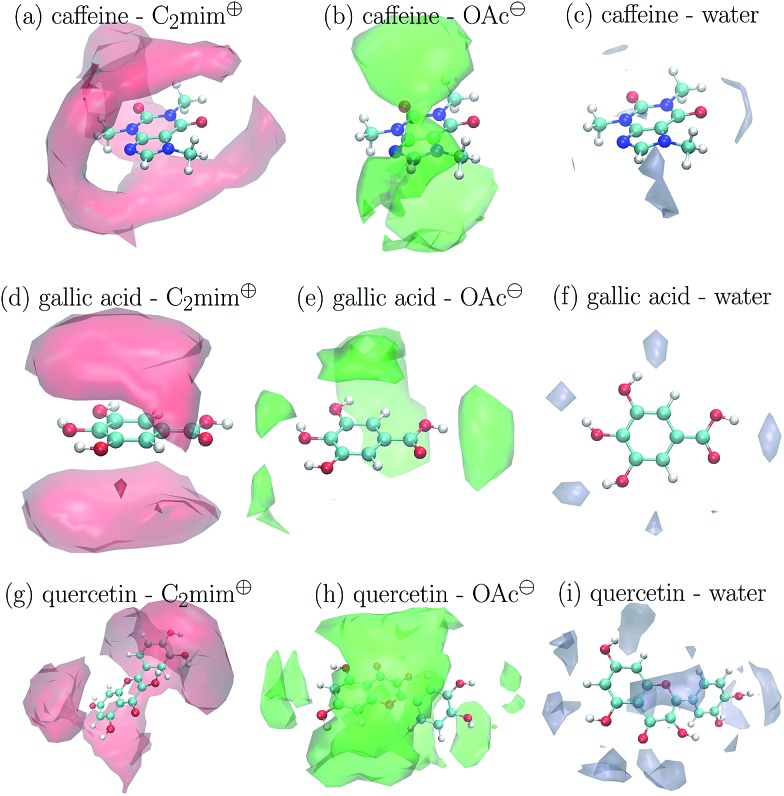
Preferred positions of the solvent molecules around the solutes at *c*_IL_ = 1.1 M.

The preferred positions of the solvent molecules in the first solvation shell are shown in Fig. 5 for *c*_IL_ = 1.1 M. Again, the Voronoi tessellation is used to restrict the displayed positions to solvent molecules which are direct neighbors of the solute to show a clearer picture. In case of caffeine in Fig. 5a and gallic acid in Fig. 5d the imidazoliums prefer positions above and below the aromatic ring systems. This is also true for the phenolic moiety of quercetin in Fig. 5g and may be explained by π–π-interactions to some extent. Acetate does not compete with C_2_mim^⊕^ on the hydrophobic positions and prefers ring positions not occupied by the cations (see Fig. 5h) or the polar hydroxyl groups of gallic acid and quercetin (see Fig. 5e and h). Here, the anions are in competition with the water molecules which accumulate only around these polar groups.

### 4.3 Hydrotropic behaviour

The formation of small micelles to cover the hydrophobic solutes seems unlikely for [C_2_mim]OAc since micellar behavior in water is only known for much longer alkyl chains of the imidazolium cations. Also the blue coordination numbers CN_H_2_O_ in Fig. 4 show that plenty of water molecules have access to the surface of the caffeine, gallic acid and quercetin even at the highest ionic liquid concentration of the aqueous mixture. Since micellar hydrotropes operate at concentrations of 1–10 mM and our investigated concentrations are much higher we exclude this possibility for C_2_mim acetate at our conditions.

**Fig. 6:**
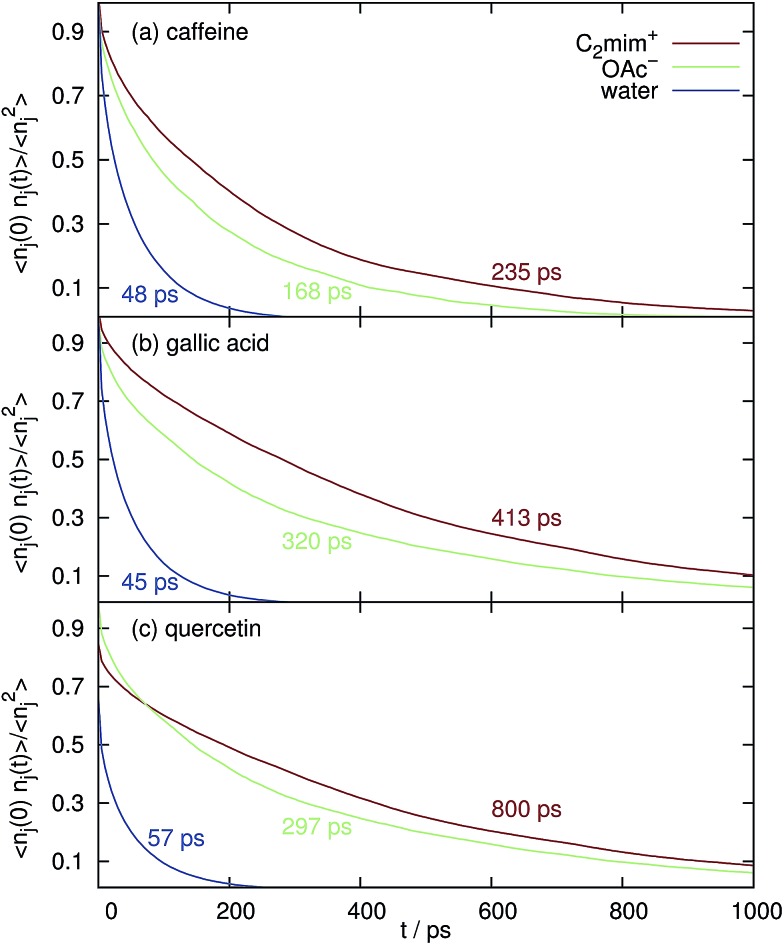
Mean residence time and the respective normalized correlation function *n*_*j*_(0)·*n*_*j*_(*t*)/*n*_*j*_^2^ of the solvent species in the first Voronoi solvation shell around the coffee solutes at *c*_IL_ = 1.1 M.

However, in Fig. 6 the normalized correlation functions *n*_*j*_(0)·*n*_*j*_(*t*)/*n*_*j*_^2^ are depicted in order to make the residence time for water and the ions comparable although their respective coordination numbers differ by an order of magnitude (see Fig. 4) at *c*_IL_ = 1.1 M. The average relaxation time of a bi-exponential fit of *n*_*j*_(0)·*n*_*j*_(*t*) is the mean residence time and is also given in the corresponding color in Fig. 6. Obviously, both cations and anions stay much longer at the solute surfaces compared to water. Therefore, the existence of “persistent” solute–ion complexes cannot be eliminated. However, these complexes do not lead to a negative free solvation energy Δ*A*(shell = 2) of water in the next solvation shell (data not shown), *i.e.* they do not promote the interaction with water.

The mean residence time of water at the surface of caffeine, gallic acid and quercetin without any ionic liquid are 31 ps, 29 ps and 35 ps, respectively, which is shorter than the values displayed in Fig. 6. However, the overwhelming part of this effect is due to the increased solvent viscosity as visible from the diffusion coefficients in Table 1.

**Fig. 7:**
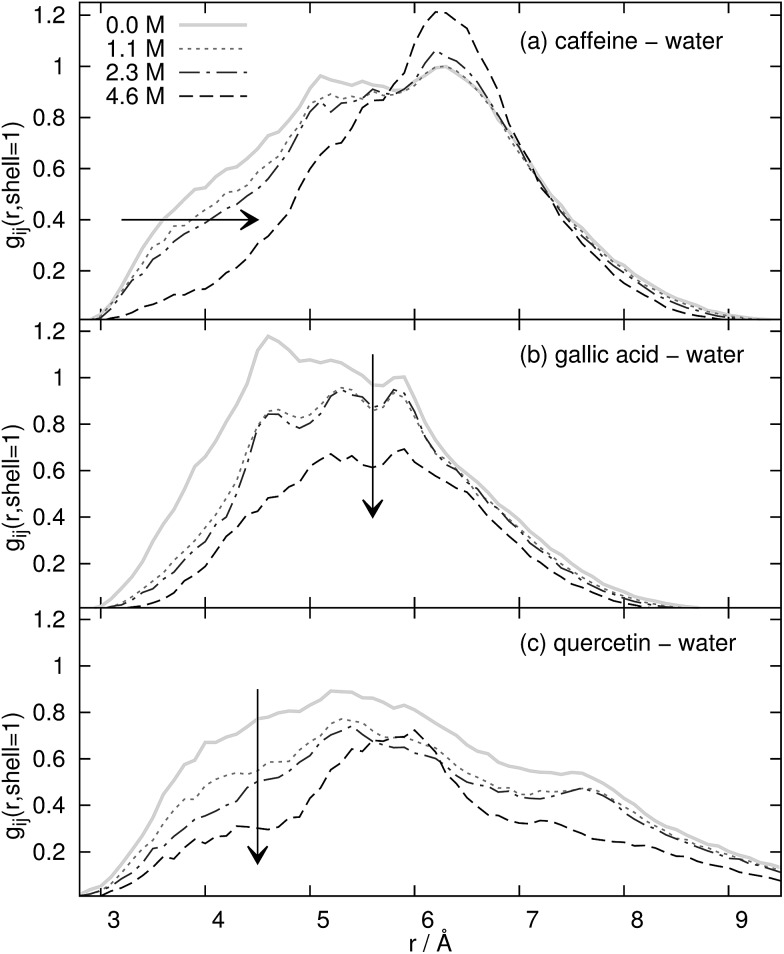
First Voronoi shell contribution to *g*_*ij*_(*r*) of water around the solutes as a function of *c*_IL_.

The last source for the hydrotropic effect is the modification of the water structure around the solute. In Fig. 7 the contribution of the first Voronoi shell to the radial distribution function *g*_*ij*_(*r*) between the coffee ingredients and water is displayed as a function of the concentration of the ionic liquid. Of course, major changes in the water structure occur between the pure water solution (solid gray line) and the most dilute solution of our ionic liquid/water mixture (dotted line). Interestingly, the water structure for all three solutes does not change very much between *c*_IL_ = 1.1 M and *c*_IL_ = 2.3 M (dash-dotted line). At the highest concentration of the aqueous ionic liquid mixture at *c*_IL_ = 4.6 M the structure changes considerably with respect to the more dilute solutions. However, two trends are visible in Fig. 7. The water at the surface of caffeine in Fig. 7a is shifted to larger distances *r*. Since these water molecules are still in the first Voronoi shell, this shift corresponds to a water displacement from the top and bottom of the caffeine molecule to the peripheric positions, *e.g.* near the oxygens. Of course, many water molecules are also expelled from the first Voronoi shell since the coordination number drops from 49.2 to 13.8. This overall water expulsion from the surface of the coffee ingredient is dominant for gallic acid and quercetin and the shift to peripheric positions plays only a minor role. The coordination number of water CN_H_2_O_ decreases to 19% of its original value for the phenolic compounds whereas still 28% of the water molecules defend their position at the surface of caffeine.

Altogether, the addition of ionic liquid leads to a dewetting of the coffee ingredient and consequently, the solubility of the solute should decrease. All three mechanisms of hydrotropes do not seem to apply for [C_2_mim]OAc in aqueous mixtures. However, if the interaction between the ionic liquid ions and the solute is strong enough, the coffee ingredient can be still extracted despite the low solubility.

### 4.4 Local interactions and extraction yields

**Fig. 8:**
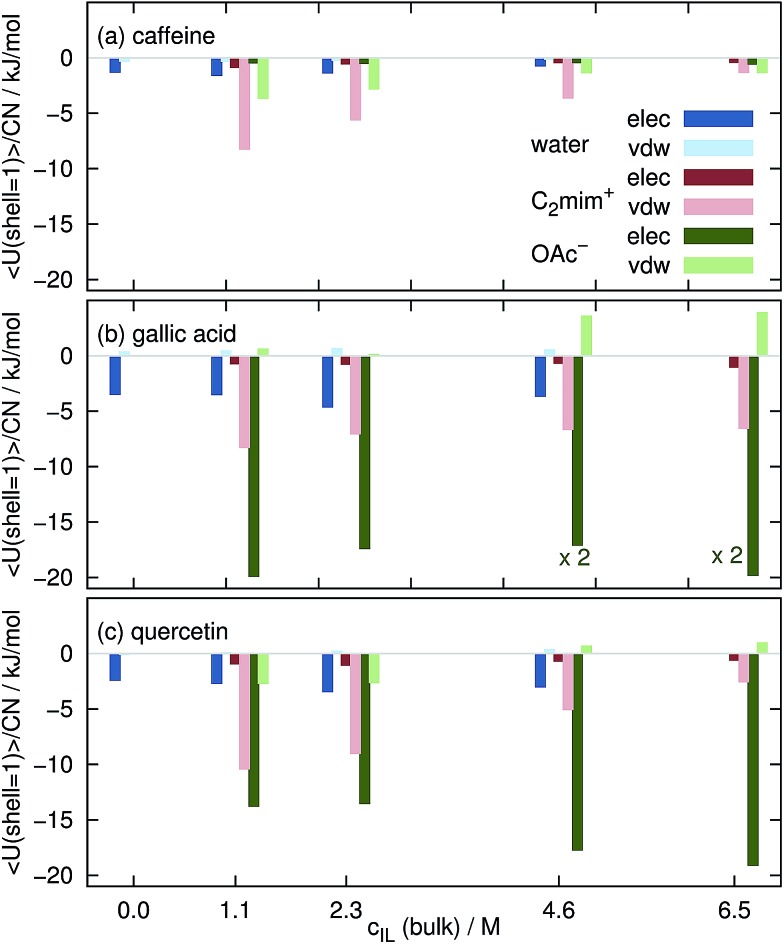
Averaged interaction energy of a water molecule (blue), a cation (red) and an anion (green) in the first solvation shell with the coffee ingredients.

The intermolecular interaction of the solutes in the aqueous mixtures in MD simulations is determined by sum of coulombic and van-der-Waals potentials. In Fig. 8 we restricted these sums to solvent molecules in the first Voronoi shell. The displayed values are averaged over the complete trajectories and divided by the respective coordination number CN_*j*_, *i.e.* they show the average interaction of one solvent molecule with the solute in the first solvation shell. Please keep in mind that these values are subject to large standard deviations of typically less than 1 kJ mol^–1^ for each water molecule and 1–5 kJ mol^–1^ per ion thus allowing only for a qualitative but not quantitative interpretation. Interactions of water, C_2_mim^⊕^ and OAc^⊖^ are in blue, red and green respectively. Dark colors denote coulombic interactions whereas bright colors van-der-Waals interactions.

Each water molecule shows stronger coulombic interactions (dark blue boxes in Fig. 8) with caffeine, gallic acid and quercetin compared to their van-der-Waals interactions. The opposite behavior is found for the imidazolium cations (bright red boxes). They mainly interact *via* van-der-Waals which may be due to their delocalized charge distribution. Since there is no particular π–π interaction in the force field, the existence of these interactions is also mainly based on van-der-Waals potentials. The situation for the anions is a little bit more complicated: the interaction with caffeine is dominated by the van-der-Waals interaction (see Fig. 8a) but turns to a dominance of coulombic interactions for gallic acid and quercetin which is most probably due to the existence of hydroxy groups in the phenolics. In a model study^76^ C_2_mim^⊕^ also showed three times larger dispersion energy with an artificial sphere compared to acetate.

However, the interaction of the ionic liquid ions with the solute per solvent molecule is at all *c*_IL_ stronger for the ions than for water explaining the expulsion of water molecules from the solute surface as observed for the free energies in Fig. 4. With increasing *c*_IL_ the molecular interaction of the cations with the solutes decreases since less favorable positions at the surface are now occupied due to the increasing numbers of cations at the surface. This trend is not true for the anions. The origin for the increased Coulomb interaction per acetate at high ionic concentrations remains unclear but maybe emerges from a rearrangement of water and anion solvation structure competing for the hydroxyl hydrogens of the phenolics. This change of solvation structure between 2.3 M < *c*_IL_<4.6 M was already observed in Fig. 7.

**Fig. 9:**
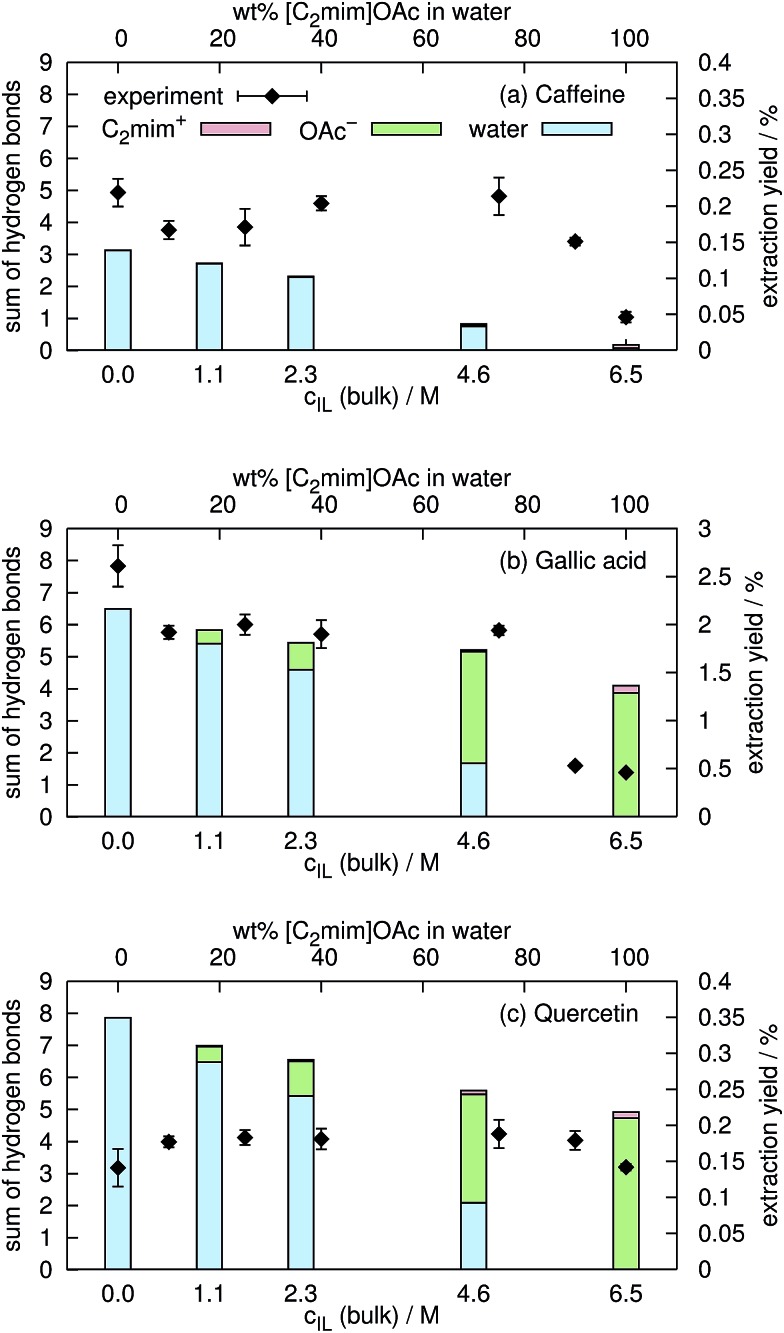
Comparison of hydrogen bonding of the solutes to water (blue), to C_2_mim^⊕^ (red) and to OAc^⊖^ (green) with respective experimental extraction yields from spent coffee. All values and their standard deviations can be found in the ESI.†

In aqueous mixtures of ionic liquids, strong hydrogen bonds between the anion and water exist.^23,72^ Nevertheless, both acetate and water, can form hydrogen bonds to the hydroxyl-hydrogens of gallic acid and quercetin. In a former publication^54^ we found that the extraction yield depends on the amount of hydrogen bonding. In the current work, we consider an interaction as hydrogen bond if the distance between the hydrogen and the electronegative atom is less than 2.4 Å and the angle between the connecting vector and the bond vector of that hydrogen is larger than 135° in agreement with ref. 77–79. In contrast, Shestopalova^13^ used a distance criterion of <2.6 Å and consequently found more hydrogen bonds. The average number of hydrogen bonds of C_2_mim^⊕^, OAc^⊖^ and water to the coffee ingredients are shown in Fig. 9 as a function of the ionic liquid concentration *c*_IL_. Below *c*_IL_ = 2.5 M, hydrogen bonds to water dominate whereas at *c*_IL_ = 4.6 M and higher the hydrogen bonds to acetate are more frequent for gallic acid and quercetin. In case of caffeine, hydrogen bonding to ionic liquid ions are negligible. Hydrogen bonds to C_2_mim^⊕^ are unlikely which is also reflected in the low coulombic interactions shown in Fig. 8. Sharma and Paul^57^ found that the number of hydrogen bonds of caffeine to water does not depend on the concentration of NaCl up to *c*_salt_ = 0.83 M. In our simulations the number of hydrogen bonds decreases by 13% while increasing *c*_IL_ from 0.0 M to 1.1 M. Obviously, sodium and chloride are not as capable as the ionic liquid to expulse water molecules from the caffeine surface.

Eventually the degree of hydrogen bonding between caffeine, gallic acid and quercetin was set in relation to the experimental extraction yields for caffeine, total phenolics and total flavonoids from spent coffee grounds (see black squares in Fig. 9). In case of caffeine and the total phenol content, the experimental extraction yield decreases with increasing ionic liquid content. This is in accordance with the computational sum of hydrogen bonds of water that is also decreasing. It seems that hydrogen bonds of these solutes to acetate are less effective indicating that water is a more suitable extraction solvent than [C_2_mim]OAc/water mixtures in this case. In contrast, the experimental extraction yield of total flavonoids does not depend on the concentration of the ionic liquid in water suggesting that the nature of the hydrogen bond partner of quercetin play a minor role.

However, one has to be careful with the comparison of experimental extraction yields and computational data of model substrates. Solubility of the hydrophobic compound and extraction yield may be affected in different ways from the solvation properties of the ionic liquid ions and water. This may also be one reason why the correlation between the hydrogen bonding and the extraction yield in this work is not as obvious as in ref. 54.

## 5 Conclusion

Traditional analysis of (un-)favorable solvation in terms of Kirkwood–Buff theory is problematic for anisotropic solute molecules using spherical integration since solvation shells are mixed up. Much more appropriate for these solutes is the decomposition of solvation shells by Voronoi tessellation. This method provides meaningful coordination numbers and volumes for each shell regardless of the solute shape.

Despite the unquestionable amphiphilic character of the ionic liquid [C_2_mim]OAc, hydrotropic behavior of this ionic liquid is not found for the solvation of the coffee ingredients caffeine, gallic acid and quercetin. Cláudio *et al.* reported a weak hydrotropic efficiency for the hydrophilic halides in case of gallic acid.^23^ Since the extraction of caffeine from guarana seeds at *c*_IL_ = 0.5 M by means of [C_2_mim]Cl and [C_2_mim]OAc in ref. 32 is very similar, the hydrotropic efficiency of [C_2_mim]OAc seems to be also low. This indicates that hydrophilic anions may not be the best choice to increase hydrotropic efficiency.

The imidazolium cations are quite effective to remove water molecules from the solute surface. Acetate and water compete for the position near the hydroxyl groups of gallic acid and quercetin. Between *c*_IL_ = 2.3 M and *c*_IL_ = 4.6 M the solvation structure around the solutes changes for the anions and water resulting in much less hydrogen bonds to water and more hydrogen bonds to acetate. For caffeine and the phenolics the experimental extraction yield decreases with decreasing number of water hydrogen bonds. In contrast, we could not detect such a trend for total flavonoid extraction. Although the increasing amount of ionic liquid in the aqueous mixture leads to a significant dewetting in our simulations, the extraction yields are much less affected which points out that solubility and extraction yield do not necessarily depend on the same solvation properties of the solvent.
