# Supplementary material for: Computational analysis of the solvation of coffee ingredients in aqueous ionic liquid mixtures
Source: RSC Adv. 2017 Jan 13;7(6):3495–504. doi: 10.1039/c6ra24736a (PMC5361174; doi:10.1039/c6ra24736a)
Supplement: Supplementary file 1 [file RA-007-C6RA24736A-s001.pdf]

Electronic Supplementary Material (ESI) for RSC Advances.
This journal is © The Royal Society of Chemistry 2016

Computational analysis of the solvation of coffee ingredients in aqueous ionic liquid mixtures

Veronika Zeindlhofer, Diana Khlan, Katharina Bica, and Christian Schröder*

Abstract

CONTENTS

Force field of the coffee ingredients 2

Force field of the solvents 16

Hydrogen bonds and extraction yields 22

FORCE FIELD OF THE COFFEE INGREDIENTS

```
*=============================================================
*. Force field of the solute species
*=============================================================
*

READ RTF CARD
*.............................................................
*. Topology of the solutes
*.............................................................
*
   99     1

MASS  101 C5A        12.011
MASS  102 C5B        12.011
MASS  103 C=C        12.011
MASS  104 C=O        12.011
MASS  105 CB         12.011
MASS  106 CR         12.011
MASS  107 HCMM        1.008
MASS  108 HOCC        1.008
MASS  109 HOCO        1.008
MASS  110 N5B        14.007
MASS  111 NC=O       14.007
MASS  112 NPYL       14.007
MASS  113 O=C        15.999
MASS  114 OR         15.999

!~~~~~~~~~~~~~~~~~~~~~~~~~~~~~~~~~~~~~~~~~~~~~~~~~~~~~~~~~~~~~
! caffeine
!~~~~~~~~~~~~~~~~~~~~~~~~~~~~~~~~~~~~~~~~~~~~~~~~~~~~~~~~~~~~~
RESIDUE CAFF  0.00000
GROUP
ATOM     N1   NPYL    0.16234
ATOM     C1     CR   -0.27493
ATOM    H11   HCMM    0.13334
ATOM    H12   HCMM     0.1324
ATOM    H13   HCMM    0.10401
ATOM     C2    C5A    0.07055
ATOM     H2   HCMM    0.12982
ATOM     N3    N5B   -0.47609
ATOM     C4    C5B    0.46692
ATOM     N5   NC=O   -0.35029
ATOM     C5     CR    0.15421
ATOM    H51   HCMM    0.02559
ATOM    H52   HCMM    0.02849
ATOM    H53   HCMM    0.00615
ATOM     C6    C=O    0.65534
ATOM     O6    O=C   -0.51422
ATOM     N7   NC=O   -0.38795
ATOM     C7     CR    0.02136
ATOM    H71   HCMM    0.05432
ATOM    H72   HCMM    0.05368
ATOM    H73   HCMM    0.05659
ATOM     C8    C=O    0.62056
```

```
ATOM      O8     O=C     -0.4932
ATOM      C9     C5A    -0.37899
BOND      C1      N1
BOND      C1     H11
BOND      C1     H12
BOND      C1     H13
BOND      N1      C9
BOND      N1      C2
BOND      C2      N3
BOND      C2      H2
BOND      N3      C4
BOND      C4      N5
BOND      C4      C9
BOND      N5      C5
BOND      C5     H51
BOND      C5     H52
BOND      C5     H53
BOND      C6      N5
BOND      C6      O6
BOND      N7      C6
BOND      N7      C7
BOND      C7     H71
BOND      C7     H72
BOND      C7     H73
BOND      C8      N7
BOND      C8      O8
BOND      C9      C8
ANGL      C1      N1      C2
ANGL      C1      N1      C9
ANGL      C2      N1      C9
ANGL      C2      N3      C4
ANGL      C4      C9      C8
ANGL      C4      C9      N1
ANGL      C4      N5      C5
ANGL      C4      N5      C6
ANGL      C5      N5      C6
ANGL      C6      N7      C7
ANGL      C6      N7      C8
ANGL      C7      N7      C8
ANGL      C8      C9      N1
ANGL      C9      C4      N3
ANGL      C9      C4      N5
ANGL      C9      C8      N7
ANGL      C9      C8      O8
ANGL     H11      C1     H12
ANGL     H11      C1     H13
ANGL     H11      C1      N1
ANGL     H12      C1     H13
ANGL     H12      C1      N1
ANGL     H13      C1      N1
ANGL      H2      C2      N1
ANGL      H2      C2      N3
ANGL     H51      C5     H52
ANGL     H51      C5     H53
ANGL     H51      C5      N5
ANGL     H52      C5     H53
ANGL     H52      C5      N5
```

ANGL	H53	C5	N5	
ANGL	H71	C7	H72	
ANGL	H71	C7	H73	
ANGL	H71	C7	N7	
ANGL	H72	C7	H73	
ANGL	H72	C7	N7	
ANGL	H73	C7	N7	
ANGL	N1	C2	N3	
ANGL	N3	C4	N5	
ANGL	N5	C6	N7	
ANGL	N5	C6	O6	
ANGL	N7	C6	O6	
ANGL	N7	C8	O8	
DIHE	C1	N1	C2	H2
DIHE	C1	N1	C2	N3
DIHE	C1	N1	C9	C4
DIHE	C1	N1	C9	C8
DIHE	C2	N1	C1	H11
DIHE	C2	N1	C1	H12
DIHE	C2	N1	C1	H13
DIHE	C2	N1	C9	C4
DIHE	C2	N1	C9	C8
DIHE	C2	N3	C4	C9
DIHE	C2	N3	C4	N5
DIHE	C4	C9	C8	N7
DIHE	C4	C9	C8	O8
DIHE	C4	N3	C2	H2
DIHE	C4	N3	C2	N1
DIHE	C4	N5	C5	H51
DIHE	C4	N5	C5	H52
DIHE	C4	N5	C5	H53
DIHE	C4	N5	C6	N7
DIHE	C4	N5	C6	O6
DIHE	C5	N5	C4	C9
DIHE	C5	N5	C4	N3
DIHE	C5	N5	C6	N7
DIHE	C5	N5	C6	O6
DIHE	C6	N5	C4	C9
DIHE	C6	N5	C4	N3
DIHE	C6	N5	C5	H51
DIHE	C6	N5	C5	H52
DIHE	C6	N5	C5	H53
DIHE	C6	N7	C7	H71
DIHE	C6	N7	C7	H72
DIHE	C6	N7	C7	H73
DIHE	C6	N7	C8	C9
DIHE	C6	N7	C8	O8
DIHE	C7	N7	C6	N5
DIHE	C7	N7	C6	O6
DIHE	C7	N7	C8	C9
DIHE	C7	N7	C8	O8
DIHE	C8	C9	C4	N3
DIHE	C8	C9	C4	N5
DIHE	C8	N7	C6	N5
DIHE	C8	N7	C6	O6
DIHE	C8	N7	C7	H71
DIHE	C8	N7	C7	H72

```
DIHE     C8     N7     C7    H73
DIHE     C9     N1     C1    H11
DIHE     C9     N1     C1    H12
DIHE     C9     N1     C1    H13
DIHE     C9     N1     C2     H2
DIHE     C9     N1     C2     N3
DIHE     N1     C9     C4     N3
DIHE     N1     C9     C4     N5
DIHE     N1     C9     C8     N7
DIHE     N1     C9     C8     O8
IMPR     N1     C2     C9     C1
IMPR     C2     N3     N1     H2
IMPR     C9     C4     N1     C8
IMPR     C8     N7     C9     O8
IMPR     N7     C6     C8     C7
IMPR     C4     C9     N3     N5
IMPR     C6     N5     N7     O6
IMPR     N5     C6     C4     C5
IMPR     C1    H11     N1    H12
IMPR     C1    H11     N1    H13
IMPR     C5    H51     N5    H52
IMPR     C5    H51     N5    H53
IMPR     C7    H71     N7    H72
IMPR     C7    H71     N7    H73
PATCHING FIRST NONE LAST NONE

!~~~~~~~~~~~~~~~~~~~~~~~~~~~~~~~~~~~~~~~~~~~~~~~~~~~~~~~~~~~~~~
! gallic acid
!~~~~~~~~~~~~~~~~~~~~~~~~~~~~~~~~~~~~~~~~~~~~~~~~~~~~~~~~~~~~~~
RESIDUE  GAL  0.00000
GROUP
ATOM     O3     OR      -0.65
ATOM     H5   HOCO        0.5
ATOM     C6    C=O     0.6338
ATOM     O4    O=C      -0.57
ATOM      C     CB     0.0862
ATOM     C1     CB      -0.15
ATOM      H   HCMM       0.15
ATOM     C2     CB     0.0825
ATOM     O2     OR    -0.5325
ATOM     H4   HOCC       0.45
ATOM     C3     CB     0.0825
ATOM     O1     OR    -0.5325
ATOM     H3   HOCC       0.45
ATOM     C4     CB     0.0825
ATOM      O     OR    -0.5325
ATOM     H2   HOCC       0.45
ATOM     C5     CB      -0.15
ATOM     H1   HCMM       0.15
BOND      C     C1
BOND      C     C5
BOND      C     C6
BOND     C1     C2
BOND     C1      H
BOND     C2     C3
BOND     C2     O2
BOND     C3     C4
```

```
BOND     C3    O1
BOND     C4    C5
BOND     C4     O
BOND     C5    H1
BOND      O    H2
BOND     O1    H3
BOND     O2    H4
BOND     C6    O3
BOND     C6    O4
BOND     O3    H5
ANGL      C    C1    C2
ANGL      C    C1     H
ANGL      C    C5    C4
ANGL      C    C5    H1
ANGL      C    C6    O3
ANGL      C    C6    O4
ANGL     C1     C    C5
ANGL     C1     C    C6
ANGL     C1    C2    C3
ANGL     C1    C2    O2
ANGL     C2    C1     H
ANGL     C2    C3    C4
ANGL     C2    C3    O1
ANGL     C2    O2    H4
ANGL     C3    C2    O2
ANGL     C3    C4    C5
ANGL     C3    C4     O
ANGL     C3    O1    H3
ANGL     C4    C3    O1
ANGL     C4    C5    H1
ANGL     C4     O    H2
ANGL     C5     C    C6
ANGL     C5    C4     O
ANGL     C6    O3    H5
ANGL     O3    C6    O4
DIHE      C    C1    C2    C3
DIHE      C    C1    C2    O2
DIHE      C    C5    C4    C3
DIHE      C    C5    C4     O
DIHE      C    C6    O3    H5
DIHE     C1     C    C5    C4
DIHE     C1     C    C5    H1
DIHE     C1     C    C6    O3
DIHE     C1     C    C6    O4
DIHE     C1    C2    C3    C4
DIHE     C1    C2    C3    O1
DIHE     C1    C2    O2    H4
DIHE     C2    C1     C    C5
DIHE     C2    C1     C    C6
DIHE     C2    C3    C4    C5
DIHE     C2    C3    C4     O
DIHE     C2    C3    O1    H3
DIHE     C3    C2    C1     H
DIHE     C3    C2    O2    H4
DIHE     C3    C4    C5    H1
DIHE     C3    C4     O    H2
DIHE     C4    C3    C2    O2
```

```
DIHE      C4      C3      O1      H3
DIHE      C4      C5       C      C6
DIHE      C5       C      C1       H
DIHE      C5       C      C6      O3
DIHE      C5       C      C6      O4
DIHE      C5      C4      C3      O1
DIHE      C5      C4       O      H2
DIHE      C6       C      C1       H
DIHE      C6       C      C5      H1
DIHE       H      C1      C2      O2
DIHE      H1      C5      C4       O
DIHE      H5      O3      C6      O4
DIHE       O      C4      C3      O1
DIHE      O1      C3      C2      O2
IMPR       C      C1      C5      C6
IMPR      C1      C2       C       H
IMPR      C2      C3      C1      O2
IMPR      C3      C4      C2      O1
IMPR      C4      C5      C3       O
IMPR      C6      O3       C      O4
IMPR      C5      C4       C      H1
PATCHING FIRST NONE LAST NONE

!~~~~~~~~~~~~~~~~~~~~~~~~~~~~~~~~~~~~~~~~~~~~~~~~~~~~~~~~~~~~~
! quercetin
!~~~~~~~~~~~~~~~~~~~~~~~~~~~~~~~~~~~~~~~~~~~~~~~~~~~~~~~~~~~~~
RESIDUE  QUE 0.0000
GROUP
ATOM      C5      CB       -0.15
ATOM      H1    HCMM        0.15
ATOM       C      CB       0.082
ATOM       O      OR      -0.532
ATOM      H2    HOCC        0.45
ATOM      C1      CB       -0.15
ATOM       H    HCMM        0.15
ATOM      C2      CB       0.082
ATOM      O1      OR      -0.532
ATOM      H3    HOCC        0.45
ATOM      C3      CB       0.086
ATOM      C4      CB       0.082
ATOM      O2      OR      -0.159
ATOM      C6     C=O       0.469
ATOM      O3     O=C      -0.569
ATOM      C7     C=C       0.091
ATOM      O4      OR      -0.526
ATOM      H4    HOCC        0.45
ATOM      C8     C=C       0.048
ATOM      C9      CB       0.028
ATOM     C10      CB       -0.15
ATOM      H5    HCMM        0.15
ATOM     C11      CB       -0.15
ATOM      H6    HCMM        0.15
ATOM     C12      CB       0.082
ATOM      O5      OR      -0.532
ATOM      H8    HOCC        0.45
ATOM     C13      CB       0.082
ATOM      O6      OR      -0.532
```

```
ATOM      H9    HOCC        0.45
ATOM     C14      CB       -0.15
ATOM      H7    HCMM        0.15
BOND       C      C1
BOND       C      C5
BOND       C       O
BOND      C1      C2
BOND      C1       H
BOND      C2      C3
BOND      C2      O1
BOND      C3      C4
BOND      C3      C6
BOND      C4      C5
BOND      C4      O2
BOND      C5      H1
BOND       O      H2
BOND      O1      H3
BOND      C6      C7
BOND      C6      O3
BOND      C7      C8
BOND      C7      O4
BOND      C8      O2
BOND      C8      C9
BOND      O4      H4
BOND      C9     C10
BOND      C9     C14
BOND     C10     C11
BOND     C10      H5
BOND     C11     C12
BOND     C11      H6
BOND     C12     C13
BOND     C12      O5
BOND     C13     C14
BOND     C13      O6
BOND     C14      H7
BOND      O5      H8
BOND      O6      H9
ANGL       C      C1      C2
ANGL       C      C1       H
ANGL       C      C5      C4
ANGL       C      C5      H1
ANGL       C       O      H2
ANGL      C1       C      C5
ANGL      C1       C       O
ANGL      C1      C2      C3
ANGL      C1      C2      O1
ANGL     C10     C11     C12
ANGL     C10     C11      H6
ANGL     C10      C9     C14
ANGL     C10      C9      C8
ANGL     C11     C10      C9
ANGL     C11     C10      H5
ANGL     C11     C12     C13
ANGL     C11     C12      O5
ANGL     C12     C11      H6
ANGL     C12     C13     C14
ANGL     C12     C13      O6
```

```
ANGL     C12      O5      H8
ANGL     C13     C12      O5
ANGL     C13     C14      C9
ANGL     C13     C14      H7
ANGL     C13      O6      H9
ANGL     C14     C13      O6
ANGL     C14      C9      C8
ANGL      C2      C1       H
ANGL      C2      C3      C4
ANGL      C2      C3      C6
ANGL      C2      O1      H3
ANGL      C3      C2      O1
ANGL      C3      C4      C5
ANGL      C3      C4      O2
ANGL      C3      C6      C7
ANGL      C3      C6      O3
ANGL      C4      C3      C6
ANGL      C4      C5      H1
ANGL      C4      O2      C8
ANGL      C5       C       O
ANGL      C5      C4      O2
ANGL      C6      C7      C8
ANGL      C6      C7      O4
ANGL      C7      C6      O3
ANGL      C7      C8      C9
ANGL      C7      C8      O2
ANGL      C7      O4      H4
ANGL      C8      C7      O4
ANGL      C9     C10      H5
ANGL      C9     C14      H7
ANGL      C9      C8      O2
DIHE       C      C1      C2      C3
DIHE       C      C1      C2      O1
DIHE       C      C5      C4      C3
DIHE       C      C5      C4      O2
DIHE      C1       C      C5      C4
DIHE      C1       C      C5      H1
DIHE      C1       C       O      H2
DIHE      C1      C2      C3      C4
DIHE      C1      C2      C3      C6
DIHE      C1      C2      O1      H3
DIHE     C10     C11     C12     C13
DIHE     C10     C11     C12      O5
DIHE     C10      C9     C14     C13
DIHE     C10      C9     C14      H7
DIHE     C10      C9      C8      C7
DIHE     C10      C9      C8      O2
DIHE     C11     C10      C9     C14
DIHE     C11     C10      C9      C8
DIHE     C11     C12     C13     C14
DIHE     C11     C12     C13      O6
DIHE     C11     C12      O5      H8
DIHE     C12     C11     C10      C9
DIHE     C12     C11     C10      H5
DIHE     C12     C13     C14      C9
DIHE     C12     C13     C14      H7
DIHE     C12     C13      O6      H9
```

DIHE	C13	C12	C11	H6
DIHE	C13	C12	O5	H8
DIHE	C13	C14	C9	C8
DIHE	C14	C13	C12	O5
DIHE	C14	C13	O6	H9
DIHE	C14	C9	C10	H5
DIHE	C14	C9	C8	C7
DIHE	C14	C9	C8	O2
DIHE	C2	C1	C	C5
DIHE	C2	C1	C	O
DIHE	C2	C3	C4	C5
DIHE	C2	C3	C4	O2
DIHE	C2	C3	C6	C7
DIHE	C2	C3	C6	O3
DIHE	C3	C2	C1	H
DIHE	C3	C2	O1	H3
DIHE	C3	C4	C5	H1
DIHE	C3	C4	O2	C8
DIHE	C3	C6	C7	C8
DIHE	C3	C6	C7	O4
DIHE	C4	C3	C2	O1
DIHE	C4	C3	C6	C7
DIHE	C4	C3	C6	O3
DIHE	C4	C5	C	O
DIHE	C4	O2	C8	C7
DIHE	C4	O2	C8	C9
DIHE	C5	C	C1	H
DIHE	C5	C	O	H2
DIHE	C5	C4	C3	C6
DIHE	C5	C4	O2	C8
DIHE	C6	C3	C2	O1
DIHE	C6	C3	C4	O2
DIHE	C6	C7	C8	C9
DIHE	C6	C7	C8	O2
DIHE	C6	C7	O4	H4
DIHE	C8	C7	C6	O3
DIHE	C8	C7	O4	H4
DIHE	C8	C9	C10	H5
DIHE	C8	C9	C14	H7
DIHE	C9	C10	C11	H6
DIHE	C9	C14	C13	O6
DIHE	C9	C8	C7	O4
DIHE	H	C1	C	O
DIHE	H	C1	C2	O1
DIHE	H1	C5	C	O
DIHE	H1	C5	C4	O2
DIHE	H5	C10	C11	H6
DIHE	H6	C11	C12	O5
DIHE	H7	C14	C13	O6
DIHE	O2	C8	C7	O4
DIHE	O3	C6	C7	O4
DIHE	O5	C12	C13	O6
IMPR	C	C1	C5	O
IMPR	C1	C2	C	H
IMPR	C2	C3	C1	O1
IMPR	C3	C4	C2	C6
IMPR	C6	C7	C3	O3

```
IMPR     C7     C8     C6     O4
IMPR     C8     C9     C7     O2
IMPR     C9    C14     C8    C10
IMPR    C10    C11     C9     H5
IMPR    C11    C12    C10     H6
IMPR    C12    C13    C11     O5
IMPR    C13    C14    C12     O6
IMPR     C5     C4      C     H1
IMPR    C14    C13     C9     H7
PATCHING FIRST NONE LAST NONE
END

READ PARA CARD FLEX
*.............................................................
*. Force field parameters of the solutes
*.............................................................
*

ATOMS
MASS  101 C5A        12.011
MASS  102 C5B        12.011
MASS  103 C=C        12.011
MASS  104 C=O        12.011
MASS  105 CB         12.011
MASS  106 CR         12.011
MASS  107 HCMM        1.008
MASS  108 HOCC        1.008
MASS  109 HOCO        1.008
MASS  110 N5B        14.007
MASS  111 NC=O       14.007
MASS  112 NPYL       14.007
MASS  113 O=C        15.999
MASS  114 OR         15.999

BONDS
!
! U_bond = k ( r - r0 )^2
!
!~~~~~~~~~~~~~~~~~~~~~~~~~~~~~~~~~~~~~~~~~~~~~~~~~~~~~~~~~~~~~~~~~~~~~~~~
!TYPE1    TYPE2    k [kcal/mol Angstroem^2]     r0 [Angstroem]
!~~~~~~~~~~~~~~~~~~~~~~~~~~~~~~~~~~~~~~~~~~~~~~~~~~~~~~~~~~~~~~~~~~~~~~~~
C5A       C5B                 512.2560              1.3770
C5A       C=O                 393.5110              1.4230
C5A       HCMM                398.0450              1.0800
C5A       N5B                 599.1910              1.3130
C5A       NPYL                453.4590              1.3640
C5B       N5B                 320.6820              1.3690
C5B       NC=O                428.3430              1.3760
C=C       C=C                 684.0390              1.3330
C=C       C=O                 328.5260              1.4680
C=C       CB                  360.3350              1.4490
C=C       OR                  397.2540              1.3730
C=O       CB                  322.9850              1.4570
C=O       NC=O                419.4910              1.3690
C=O       O=C                 931.9630              1.2220
C=O       OR                  417.4760              1.3550
CB        CB                  401.0680              1.3740
```

```
CB        HCMM              381.8530              1.0840
CB        OR                404.0190              1.3760
CR        HCMM              342.9910              1.0930
CR        NC=O              335.6510              1.4360
CR        NPYL              440.0020              1.4450
HOCC      OR                564.1430              0.9730
HOCO      OR                532.7660              0.9810

ANGLES
!
! U_angle = k ( theta - theta0 )^2
!
!~~~~~~~~~~~~~~~~~~~~~~~~~~~~~~~~~~~~~~~~~~~~~~~~~~~~~~~~~~~~~~~~~~~~~~~~~~~
!TYPE1    TYPE2     TYPE3     k [kcal/mol rad^2]     theta0 [deg]
!~~~~~~~~~~~~~~~~~~~~~~~~~~~~~~~~~~~~~~~~~~~~~~~~~~~~~~~~~~~~~~~~~~~~~~~~~~~
C5A       C5B       N5B              74.7000            111.6000
C5A       C5B       NC=O             67.4320            123.7000
C5A       C=O       NC=O             77.3630            114.6000
C5A       C=O       O=C              74.5560            126.5000
C5A       N5B       C5B              86.7910            103.8000
C5A       NPYL      C5A              82.9040            109.6000
C5A       NPYL      CR               61.4590            123.4000
C5B       C5A       C=O              55.1260            130.1000
C5B       C5A       NPYL             58.5080            107.3000
C5B       NC=O      C=O              75.4200            117.6000
C5B       NC=O      CR               69.0870            121.3000
C=C       C=C       C=O              39.2210            111.3000
C=C       C=C       CB               43.0350            117.5000
C=C       C=C       OR               80.3860            121.3000
C=C       C=O       CB               70.0230            112.9000
C=C       C=O       O=C              67.3600            122.6000
C=C       CB        CB               51.2400            119.7000
C=C       OR        CB              102.0470            110.7000
C=C       OR        HOCC             58.7240            105.7000
C=O       C5A       NPYL             64.7690            125.4000
C=O       C=C       OR               82.1850            116.7000
C=O       CB        CB               57.4290            114.5000
C=O       NC=O      C=O              51.0240            120.3000
C=O       NC=O      CR               59.0840            119.6000
C=O       OR        HOCO             41.9560            111.9000
CB        C=C       OR               86.2150            114.4000
CB        C=O       O=C              52.8230            120.0000
CB        C=O       OR               58.1480            102.9000
CB        CB        CB               48.1450            120.0000
CB        CB        HCMM             40.5170            120.6000
CB        CB        OR               69.6630            116.5000
CB        OR        HOCC             52.2470            105.4000
HCMM      C5A       N5B              46.2740            125.1000
HCMM      C5A       NPYL             44.4030            121.1000
HCMM      CR        HCMM             37.1340            108.8000
HCMM      CR        NC=O             53.2550            107.6000
HCMM      CR        NPYL             58.3640            106.3000
N5B       C5A       NPYL             72.8290            110.9000
N5B       C5B       NC=O             76.6430            121.1000
NC=O      C=O       NC=O            116.0090            114.9000
NC=O      C=O       O=C              65.2730            127.2000
O=C       C=O       OR               83.1200            124.4000
```

```
DIHEDRALS
!
! U_dihedral = k ( 1 + Cos[n phi - delta] )
!
!~~~~~~~~~~~~~~~~~~~~~~~~~~~~~~~~~~~~~~~~~~~~~~~~~~~~~~~~~~~~~~~~~~~~~
!TYPE1    TYPE2     TYPE3     TYPE4    k [kcal/mol]    n     delta [deg]
!~~~~~~~~~~~~~~~~~~~~~~~~~~~~~~~~~~~~~~~~~~~~~~~~~~~~~~~~~~~~~~~~~~~~~
C5A       C5B       N5B       C5A           3.5000     2       180.0000
C5A       C5B       NC=O      C=O           3.0000     2       180.0000
C5A       C5B       NC=O      CR            3.0000     2       180.0000
C5A       C=O       NC=O      C=O           3.0000     2       180.0000
C5A       C=O       NC=O      CR            3.0000     2       180.0000
C5A       N5B       C5B       NC=O          3.5000     2       180.0000
C5A       NPYL      C5A       C5B           2.0000     2       180.0000
C5A       NPYL      C5A       C=O           2.0000     2       180.0000
C5A       NPYL      C5A       HCMM          2.0000     2       180.0000
C5A       NPYL      C5A       N5B           2.0000     2       180.0000
C5A       NPYL      CR        HCMM         -0.0570     3         0.0000
C5B       C5A       C=O       NC=O          1.2500     2       180.0000
C5B       C5A       C=O       O=C           1.2500     2       180.0000
C5B       C5A       NPYL      CR            2.0000     2       180.0000
C5B       N5B       C5A       HCMM          3.5000     2       180.0000
C5B       N5B       C5A       NPYL          3.5000     2       180.0000
C5B       NC=O      C=O       NC=O          3.0000     2       180.0000
C5B       NC=O      C=O       O=C           3.0000     2       180.0000
C5B       NC=O      CR        HCMM          0.1500     3         0.0000
C=C       C=C       C=O       CB            1.2500     2       180.0000
C=C       C=C       C=O       O=C           1.2500     2       180.0000
C=C       C=C       CB        CB            0.2170     3         0.0000
C=C       C=C       CB        CB            0.7710     2       180.0000
C=C       C=C       OR        CB            1.5500     2       180.0000
C=C       C=C       OR        HOCC          1.4050     2       180.0000
C=C       C=C       OR        HOCC         -0.1070     1         0.0000
C=C       C=C       OR        HOCC         -0.2280     3         0.0000
C=C       C=O       CB        CB            1.2500     2       180.0000
C=C       CB        CB        CB            1.0000     2       180.0000
C=C       CB        CB        HCMM          1.0000     2       180.0000
C=C       OR        CB        CB            1.6000     2       180.0000
C=O       C5A       C5B       N5B           3.5000     2       180.0000
C=O       C5A       C5B       NC=O          0.0000     1         0.0000
C=O       C5A       NPYL      CR            3.0000     2       180.0000
C=O       C=C       C=C       CB            0.9000     2       180.0000
C=O       C=C       C=C       OR            0.9000     2       180.0000
C=O       C=C       OR        HOCC          1.8000     2       180.0000
C=O       CB        CB        CB            3.5000     2       180.0000
C=O       CB        CB        HCMM          1.0000     2       180.0000
C=O       CB        CB        OR            1.0000     2       180.0000
C=O       NC=O      C5B       N5B           3.0000     2       180.0000
C=O       NC=O      C=O       NC=O          3.0000     2       180.0000
C=O       NC=O      C=O       O=C           0.3880     1         0.0000
C=O       NC=O      C=O       O=C          -0.0720     3         0.0000
C=O       NC=O      C=O       O=C          -0.2920     2       180.0000
C=O       NC=O      CR        HCMM          0.0110     3         0.0000
C=O       NC=O      CR        HCMM          0.6810     2       180.0000
C=O       NC=O      CR        HCMM         -1.0500     1         0.0000
CB        C=C       C=C       OR            0.9000     2       180.0000
```

```
CB        C=C       OR        CB             1.5500     2       180.0000
CB        C=O       C=C       OR             1.2500     2       180.0000
CB        C=O       OR        HOCO           1.9460     2       180.0000
CB        C=O       OR        HOCO          -0.0470     3         0.0000
CB        CB        C=C       OR             1.0000     2       180.0000
CB        CB        C=O       O=C            1.1280     2       180.0000
CB        CB        C=O       OR             0.8710     2       180.0000
CB        CB        CB        CB             3.5000     2       180.0000
CB        CB        CB        HCMM           3.5000     2       180.0000
CB        CB        CB        OR             3.5000     2       180.0000
CB        CB        OR        HOCC           1.4010     2       180.0000
CR        NC=O      C5B       N5B            3.0000     2       180.0000
CR        NC=O      C=O       NC=O           3.0000     2       180.0000
CR        NC=O      C=O       O=C            3.1470     2       180.0000
CR        NC=O      C=O       O=C           -0.0730     3         0.0000
CR        NC=O      C=O       O=C           -0.1600     1         0.0000
CR        NPYL      C5A       HCMM           2.0000     2       180.0000
CR        NPYL      C5A       N5B            2.0000     2       180.0000
HCMM      CB        CB        HCMM           3.5000     2       180.0000
HCMM      CB        CB        OR             3.5000     2       180.0000
HOCO      OR        C=O       O=C            0.8310     1         0.0000
HOCO      OR        C=O       O=C            3.0760     2       180.0000
HOCO      OR        C=O       O=C           -0.0290     3         0.0000
N5B       C5B       C5A       NPYL           3.5000     2       180.0000
NC=O      C5B       C5A       NPYL           3.5000     2       180.0000
NC=O      C=O       C5A       NPYL           1.2500     2       180.0000
NPYL      C5A       C=O       O=C            1.2500     2       180.0000
O=C       C=O       C=C       OR             1.2500     2       180.0000
OR        C=C       C=C       OR             6.0000     2       180.0000
OR        CB        CB        OR             3.5000     2       180.0000

IMPROPERS
! n > 0:
! U_improper = k ( 1 + Cos[n phi - delta] )
!
! n = 0:
! U_improper = k ( phi - delta )^2
!
!~~~~~~~~~~~~~~~~~~~~~~~~~~~~~~~~~~~~~~~~~~~~~~~~~~~~~~~~~~~~~~~~~~~~~~~~~~~~~~~~

!TYPE1    TYPE2     TYPE3     TYPE4    k [kcal/mol]    n     delta [deg]
!~~~~~~~~~~~~~~~~~~~~~~~~~~~~~~~~~~~~~~~~~~~~~~~~~~~~~~~~~~~~~~~~~~~~~~~~~~~~~~~~

C5A       C5B       NPYL      C=O            3.5980     0.0         0.0000
C5A       N5B       NPYL      HCMM           4.8940     0.0         0.0000
C5B       C5A       N5B       NC=O           2.8790     0.0         0.0000
C=C       C=C       C=O       OR             1.4390     0.0         0.0000
C=C       CB        C=C       OR             1.4390     0.0         0.0000
C=O       C=C       CB        O=C            9.3560     0.0         0.0000
C=O       NC=O      C5A       O=C            9.3560     0.0         0.0000
C=O       NC=O      NC=O      O=C            8.1320     0.0         0.0000
C=O       OR        CB        O=C            9.1400     0.0         0.0000
CB        CB        C=C       CB             2.2310     0.0         0.0000
CB        CB        CB        C=O            1.9430     0.0         0.0000
CB        CB        CB        HCMM           1.0790     0.0         0.0000
CB        CB        CB        OR             3.4540     0.0         0.0000
CR        HCMM      NC=O      HCMM           0.0000     0.0         0.0000
```

```
CR        HCMM      NPYL      HCMM          0.0000     0.0         0.0000
NC=O      C=O       C5B       CR           -1.4390     0.0         0.0000
NC=O      C=O       C=O       CR           -1.4390     0.0         0.0000
NPYL      C5A       C5A       CR            0.8640     0.0         0.0000

NONBONDED NBXMOD 5
!~~~~~~~~~~~~~~~~~~~~~~~~~~~~~~~~~~~~~~~~~~~~~~~~~~~~~~~~~~~~~~~~~~~~~~~~
!TYPE1          epsilon    rmin/2       epsilon14   rmin14/2
!           [kcal/mol] [Angstroem]    [kcal/mol] [Angstroem]
!~~~~~~~~~~~~~~~~~~~~~~~~~~~~~~~~~~~~~~~~~~~~~~~~~~~~~~~~~~~~~~~~~~~~~~~~
C5A     0.00   -0.05000   2.04000
C5B     0.00   -0.05000   2.04000
C=C     0.00   -0.06800   2.09000
C=O     0.00   -0.11000   2.00000
CB      0.00   -0.07000   1.99240
CR      0.00   -0.05500   2.17500 0.00   -0.01000   2.17500
HCMM    0.00   -0.02200   1.32000
HOCC    0.00   -0.04600   0.22450
HOCO    0.00   -0.04600   0.22450
N5B     0.00   -0.20000   1.85000
NC=O    0.00   -0.20000   1.85000
NPYL    0.00   -0.09000   1.72000
O=C     0.00   -0.12000   1.70000
OR      0.00   -0.15210   1.77000
END
```

FORCE FIELD OF THE SOLVENTS

```
*===========================================================
*. Force field of the solvent species
*===========================================================
*

READ RTF CARD
*...........................................................
*. Topology of the solvents
*...........................................................
*
   99      1

MASS      1 H1       1.008000
MASS      2 H4       1.008000
MASS      3 H5       1.008000
MASS      4 HC       1.008000
MASS      5 HT       1.008000
MASS      6 C3      12.010000
MASS      7 CC      12.010000
MASS      8 CD      12.010000
MASS      9 CT      12.010000
MASS     10 C       12.010000
MASS     11 NA      14.010000
MASS     12 OA      16.000000
MASS     13 OT      16.000000

!~~~~~~~~~~~~~~~~~~~~~~~~~~~~~~~~~~~~~~~~~~~~~~~~~~~~~~~~~~~
! water
!~~~~~~~~~~~~~~~~~~~~~~~~~~~~~~~~~~~~~~~~~~~~~~~~~~~~~~~~~~~
RESIDUE SPCE       0.000 ! spce water model, generate using noangle
   nodihedral
GROUP
ATOM OH2  OT     -0.848
ATOM H1   HT      0.424
ATOM H2   HT      0.424
BOND OH2 H1 OH2 H2 H1 H2     ! the last bond is needed for shake
ANGLE H1 OH2 H2              ! required
DONOR H1 OH2
DONOR H2 OH2
ACCEPTOR OH2
PATCHING FIRS NONE LAST NONE

!~~~~~~~~~~~~~~~~~~~~~~~~~~~~~~~~~~~~~~~~~~~~~~~~~~~~~~~~~~~
!EMIM
!~~~~~~~~~~~~~~~~~~~~~~~~~~~~~~~~~~~~~~~~~~~~~~~~~~~~~~~~~~~
RESIDUE EMIM  1.000
GROUP
ATOM C    C3      -0.110 !          H
ATOM H    H1       0.117 !          |
ATOM H1   H1       0.117 !      H1--C--H2
ATOM H2   H1       0.117 !          |
ATOM N    NA       0.085 !          N1
ATOM C1   CC      -0.027 !        //   \
ATOM H3   H5       0.230 !       //     C2--H4
```

```
ATOM C2   CC      -0.189 !   H3--C1     ||
ATOM H4   H4       0.253 !        \      C3--H5
ATOM N1   NA       0.085 !         \    /
ATOM C3   CD      -0.189 !            N
ATOM H5   H4       0.253 !            |
ATOM C4   C3      -0.043 !       H6--C4--H7
ATOM H6   H1       0.105 !            |
ATOM H7   H1       0.105 !       H8--C5--H9
ATOM C5   C3      -0.107 !            |
ATOM H8   HC       0.066 !            H10
ATOM H9   HC       0.066 !
ATOM H10  HC       0.066 !
BOND C     H                    ! dist      1.0796
BOND C     H1                   ! dist      1.0785
BOND C     H2                   ! dist      1.0794
BOND C     N1                   ! dist      1.4658
BOND N     C1                   ! dist      1.3138
BOND N     C3                   ! dist      1.3781
BOND N     C4                   ! dist      1.4777
BOND C1    N1                   ! dist      1.3157
BOND C1    H3                   ! dist      1.0692
BOND N1    C2                   ! dist      1.3785
BOND C2    C3                   ! dist      1.3416
BOND C2    H4                   ! dist      1.0679
BOND C3    H5                   ! dist      1.0688
BOND C4    H6                   ! dist      1.0814
BOND C4    H7                   ! dist      1.0798
BOND C4    C5                   ! dist      1.5218
BOND C5    H8                   ! dist      1.0835
BOND C5    H9                   ! dist      1.0834
BOND C5    H10                  ! dist      1.0837
ANGL C     N1    C1             ! angle   126.4111
ANGL C     N1    C2             ! angle   125.6300
ANGL H     C     H1             ! angle   109.5510
ANGL H     C     H2             ! angle   110.2559
ANGL H     C     N1             ! angle   109.2611
ANGL H1    C     H2             ! angle   109.5172
ANGL H1    C     N1             ! angle   108.8792
ANGL H2    C     N1             ! angle   109.3538
ANGL N     C1    N1             ! angle   109.8941
ANGL N     C1    H3             ! angle   125.1030
ANGL N     C3    C2             ! angle   107.1959
ANGL N     C3    H5             ! angle   122.1025
ANGL N     C4    H6             ! angle   106.7043
ANGL N     C4    H7             ! angle   106.9082
ANGL N     C4    C5             ! angle   112.0836
ANGL C1    N     C3             ! angle   107.9285
ANGL C1    N     C4             ! angle   126.1921
ANGL C1    N1    C2             ! angle   107.9577
ANGL N1    C1    H3             ! angle   125.0025
ANGL N1    C2    C3             ! angle   107.0236
ANGL N1    C2    H4             ! angle   122.0406
ANGL C2    C3    H5             ! angle   130.7003
ANGL C3    N     C4             ! angle   125.8669
ANGL C3    C2    H4             ! angle   130.9352
ANGL C4    C5    H8             ! angle   111.2780
ANGL C4    C5    H9             ! angle   111.5813
```

```
ANGL C4    C5    H10               ! angle   109.0336
ANGL H6    C4    H7                ! angle   107.7086
ANGL H6    C4    C5                ! angle   111.3594
ANGL H7    C4    C5                ! angle   111.7864
ANGL H8    C5    H9                ! angle   108.5717
ANGL H8    C5    H10               ! angle   108.3168
ANGL H9    C5    H10               ! angle   107.9497
DIHE H     C     N1    C1          ! dihe   -120.0991
DIHE H1    C     N1    C1          ! dihe     -0.4861
DIHE H2    C     N1    C1          ! dihe    119.1407
DIHE H     C     N1    C2          ! dihe     60.3500
DIHE H1    C     N1    C2          ! dihe    179.9630
DIHE H2    C     N1    C2          ! dihe    -60.4102
DIHE C3    N     C1    N1          ! dihe     -0.0723
DIHE C4    N     C1    N1          ! dihe   -178.8479
DIHE C3    N     C1    H3          ! dihe   -179.8641
DIHE C4    N     C1    H3          ! dihe      1.3603
DIHE C1    N     C3    C2          ! dihe      0.1214
DIHE C4    N     C3    C2          ! dihe    178.9021
DIHE C1    N     C3    H5          ! dihe    179.7449
DIHE C4    N     C3    H5          ! dihe     -1.4744
DIHE C1    N     C4    H6          ! dihe    -17.3257
DIHE C3    N     C4    H6          ! dihe    164.1119
DIHE C1    N     C4    H7          ! dihe   -132.3592
DIHE C3    N     C4    H7          ! dihe     49.0783
DIHE C1    N     C4    C5          ! dihe    104.8236
DIHE C3    N     C4    C5          ! dihe    -73.7389
DIHE N     C1    N1    C           ! dihe   -179.6202
DIHE H3    C1    N1    C           ! dihe      0.1719
DIHE N     C1    N1    C2          ! dihe     -0.0039
DIHE H3    C1    N1    C2          ! dihe    179.7882
DIHE C     N1    C2    C3          ! dihe    179.7003
DIHE C1    N1    C2    C3          ! dihe      0.0802
DIHE C     N1    C2    H4          ! dihe     -0.0517
DIHE C1    N1    C2    H4          ! dihe   -179.6718
DIHE N1    C2    C3    N           ! dihe     -0.1217
DIHE H4    C2    C3    N           ! dihe    179.6001
DIHE N1    C2    C3    H5          ! dihe   -179.7009
DIHE H4    C2    C3    H5          ! dihe      0.0208
DIHE N     C4    C5    H8          ! dihe    -60.5075
DIHE H6    C4    C5    H8          ! dihe     58.9472
DIHE H7    C4    C5    H8          ! dihe    179.4809
DIHE N     C4    C5    H9          ! dihe     60.9322
DIHE H6    C4    C5    H9          ! dihe   -179.6130
DIHE H7    C4    C5    H9          ! dihe    -59.0794
DIHE N     C4    C5    H10         ! dihe   -179.9216
DIHE H6    C4    C5    H10         ! dihe    -60.4668
DIHE H7    C4    C5    H10         ! dihe     60.0669
IMPH C4    C1    N     C3
IMPH H3    N1    C1    N
IMPH C     C1    N1    C2
IMPH C3    H4    C2    N1
IMPH C2    H5    C3    N
DONOR  H3  C1
DONOR  H4  C2
DONOR  H5  C3
PATCH FIRST NONE LAST NONE
```

```
!~~~~~~~~~~~~~~~~~~~~~~~~~~~~~~~~~~~~~~~~~~~~~~~~~~~~~~~~~~~~~~
! acetate
!~~~~~~~~~~~~~~~~~~~~~~~~~~~~~~~~~~~~~~~~~~~~~~~~~~~~~~~~~~~~~~
RESIDUE OAC -1.000
GROUP
ATOM C     C3       -0.218 !      H2    O
ATOM H     HC        0.010 !      |    /
ATOM H1    HC        0.010 !  H--C---C1
ATOM H2    HC        0.010 !      |    \
ATOM C1    C         0.774 !      H1    O1
ATOM O     OA       -0.793 !
ATOM O1    OA       -0.793 !
BOND C      H                    ! dist       1.0885
BOND C      H1                   ! dist       1.0890
BOND C      H2                   ! dist       1.0858
BOND C      C1                   ! dist       1.5538
BOND C1     O                    ! dist       1.2333
BOND C1     O1                   ! dist       1.2354
ANGL C      C1     O             ! angle    116.0140
ANGL C      C1     O1            ! angle    114.5436
ANGL H      C      H1            ! angle    106.6972
ANGL H      C      H2            ! angle    109.2573
ANGL H      C      C1            ! angle    109.6829
ANGL H1     C      H2            ! angle    109.2436
ANGL H1     C      C1            ! angle    109.7254
ANGL H2     C      C1            ! angle    112.0849
ANGL O      C1     O1            ! angle    129.4425
DIHE H      C      C1    O       ! dihe    -121.4873
DIHE H1     C      C1    O       ! dihe     121.6133
DIHE H2     C      C1    O       ! dihe       0.0569
DIHE H      C      C1    O1      ! dihe      58.5127
DIHE H1     C      C1    O1      ! dihe     -58.3867
DIHE H2     C      C1    O1      ! dihe    -179.9431
IMPH C      O      C1    O1
ACCEPTOR   O  C1
ACCEPTOR  O1  C1
PATCH FIRST NONE LAST NONE
END


READ PARA CARD FLEX
*............................................................
*. Force field parameters of the solvents
*............................................................
*

ATOMS
MASS      1 H1       1.008000
MASS      2 H4       1.008000
MASS      3 H5       1.008000
MASS      4 HC       1.008000
MASS      5 HT       1.008000
MASS      6 C3      12.010000
MASS      7 CC      12.010000
MASS      8 CD      12.010000
MASS      9 CT      12.010000
```

```
MASS    10 C      12.010000
MASS    11 NA     14.010000
MASS    12 OA     16.000000
MASS    13 OT     16.000000

BONDS
!
! U_bond = k ( r - r0 )^2
!
!~~~~~~~~~~~~~~~~~~~~~~~~~~~~~~~~~~~~~~~~~~~~~~~~~~~~~~~~~~~~~~~~~~~~~~~~
!TYPE1    TYPE2    k [kcal/mol Angstroem^2]     r0 [Angstroem]
!~~~~~~~~~~~~~~~~~~~~~~~~~~~~~~~~~~~~~~~~~~~~~~~~~~~~~~~~~~~~~~~~~~~~~~~~
HT        HT                 0.0                 1.632980862
HT        OT               450.0                 1.0000
C3        H1               335.90                1.093
C3        NA               334.70                1.456
CC        NA               438.80                1.371
CD        NA               438.80                1.371
CC        H5               356.00                1.079
CC        CD               504.00                1.371
CC        H4               350.10                1.083
CD        H4               350.10                1.083
C3        C3               303.10                1.535
C3        HC               337.30                1.092
C         C3               328.30                1.508
C         OA               648.00                1.214

ANGLES
!
! U_angle = k ( theta - theta0 )^2
!
!~~~~~~~~~~~~~~~~~~~~~~~~~~~~~~~~~~~~~~~~~~~~~~~~~~~~~~~~~~~~~~~~~~~~~~~~
!TYPE1    TYPE2    TYPE3    k [kcal/mol rad^2]    theta0 [deg]
!~~~~~~~~~~~~~~~~~~~~~~~~~~~~~~~~~~~~~~~~~~~~~~~~~~~~~~~~~~~~~~~~~~~~~~~~
HT        OT       HT          55.0               109.4667
C3        NA       CC          62.560             125.090
H1        C3       H1          39.180             109.550
H1        C3       NA          49.900             109.450
NA        CC       NA          73.650             109.330   ! SAME AS
   NA C2 NA
NA        CC       H5          49.760             122.100
NA        CD       CC          72.910             109.420
NA        CD       H4          50.220             119.660
NA        C3       C3          65.730             112.810
CC        NA       CD          63.880             128.010
CC        NA       CC          68.940             109.900
NA        CC       CD          72.910             109.420
NA        CC       H4          50.220             119.660
CC        CD       H4          47.190             129.110
CD        NA       C3          62.560             125.090
CD        CC       H4          47.190             129.110
C3        C3       HC          46.370             110.050
H1        C3       C3          46.360             110.070
HC        C3       HC          39.430             108.350
C3        C        OA          68.030             123.110
HC        C3       C           47.200             109.680
OA        C        OA          78.170             130.380
```

```
DIHEDRALS
!
! U_dihedral = k ( 1 + Cos[n phi - delta] )
!
!~~~~~~~~~~~~~~~~~~~~~~~~~~~~~~~~~~~~~~~~~~~~~~~~~~~~~~~~~~~~~~~~~~~~~~~~~~~~~~
!TYPE1    TYPE2     TYPE3     TYPE4    k [kcal/mol]    n     delta [deg]
!~~~~~~~~~~~~~~~~~~~~~~~~~~~~~~~~~~~~~~~~~~~~~~~~~~~~~~~~~~~~~~~~~~~~~~~~~~~~~~
X         C3        NA        X           0.000        2       0.0
X         CC        NA        X           1.700        2     180.0
X         CD        NA        X           1.700        2     180.0
X         CC        CD        X           4.000        2     180.0
X         C3        C3        X           0.156        3       0.0
CC        NA        C3        C3          0.049        1       0.0
CC        NA        C3        C3          0.284        2     180.0
CC        NA        C3        C3          0.301        3     180.0
CC        NA        C3        C3          0.025        6       0.0
X         C         C3        X           0.000        2     180.0
HC        C3        C         OA          0.800        1       0.0
HC        C3        C         OA          0.080        3     180.0

IMPROPERS
! n > 0:
! U_improper = k ( 1 + Cos[n phi - delta] )
!
! n = 0:
! U_improper = k ( phi - delta )^2
!
!~~~~~~~~~~~~~~~~~~~~~~~~~~~~~~~~~~~~~~~~~~~~~~~~~~~~~~~~~~~~~~~~~~~~~~~~~~~~~~~~~~~~~~~~~~~

!TYPE1    TYPE2     TYPE3     TYPE4    k [kcal/mol]    n     delta [deg]
!~~~~~~~~~~~~~~~~~~~~~~~~~~~~~~~~~~~~~~~~~~~~~~~~~~~~~~~~~~~~~~~~~~~~~~~~~~~~~~~~~~~~~~~~~~~


C3        CC        NA        CD        1.100          2     180.0
H5        NA        CC        NA        1.100          2     180.0
C3        CC        NA        CC        1.100          2     180.0
CD        H4        CC        NA        1.100          2     180.0
CC        H4        CD        NA        1.100          2     180.0

NONBONDED NBXMOD 5
!~~~~~~~~~~~~~~~~~~~~~~~~~~~~~~~~~~~~~~~~~~~~~~~~~~~~~~~~~~~~~~~~~~~~~~~~~~~~~~
!TYPE1          epsilon    rmin/2       epsilon14   rmin14/2
!           [kcal/mol] [Angstroem]    [kcal/mol] [Angstroem]
!~~~~~~~~~~~~~~~~~~~~~~~~~~~~~~~~~~~~~~~~~~~~~~~~~~~~~~~~~~~~~~~~~~~~~~~~~~~~~~
HT       0.00   -0.0000    0.0000
OT       0.00   -0.15210   1.7770
C3       0.00   -0.1094    1.9080      0.00   -0.0547    1.9080
H1       0.00   -0.0157    1.3870      0.00   -0.00775   1.3870
NA       0.00   -0.1700    1.8240      0.00   -0.0850    1.8240
CC       0.00   -0.0860    1.9080      0.00   -0.0430    1.9080
CD       0.00   -0.0860    1.9080      0.00   -0.0430    1.9080
H5       0.00   -0.0150    1.3590      0.00   -0.0075    1.3590
H4       0.00   -0.0150    1.4090      0.00   -0.0075    1.4090
HC       0.00   -0.0157    1.4870      0.00   -0.00775   1.4870
C        0.00   -0.0860    1.9080      0.00   -0.0430    1.9080
OA       0.00   -0.2100    1.6612      0.00   -0.1050    1.6612
END
```

HYDROGEN BONDS AND EXTRACTION YIELDS

	c_{IL} [% wt]	HBONDS<br>water	 C_2mim^+	 OAc^-	extraction yield<br>[%]
caffeine					
	0	3.1±1.0			0.219±0.019
	10				0.167±0.013
	18	2.7±1.0	0.0±0.1	0.0±0.1	
	25				0.171±0.026
	36	2.3±1.0	0.0±0.1	0.0±0.1	
	40				0.204±0.010
	70	0.8±0.8	0.1±0.2	0.0±0.2	
	75				0.214±0.026
	90				0.151±0.005
	100		0.1±0.3	0.1±0.3	0.046±0.007
gallic acid					
	0	6.5±1.5			0.261±0.043
	10				0.192±0.014
	18	5.4±1.5	0.0±0.1	0.4±0.6	
	25				0.200±0.021
	36	4.6±1.4	0.0±0.2	0.9±0.6	
	40				0.190±0.029
	70	1.7±1.1	0.1±0.2	3.5±0.8	
	75				0.194±0.010
	90				0.053±0.005
	100		0.2±0.5	3.9±0.5	0.046±0.000
quercetin					
	0	7.9±1.7			0.141±0.026
	10				0.177±0.008
	18	6.5±1.7	0.0±0.2	0.5±0.6	
	25				0.183±0.010
	36	5.4±1.6	0.1±0.2	1.1±0.8	
	40				0.181±0.014
	70	2.1±1.2	0.1±0.4	3.4±0.8	
	75				0.188±0.020
	90				0.179±0.013
	100		0.2±0.4	4.7±0.6	0.142±0.005
